# Role of N343 glycosylation on the SARS-CoV-2 S RBD structure and co-receptor binding across variants of concern

**DOI:** 10.7554/eLife.95708

**Published:** 2024-06-12

**Authors:** Callum M Ives, Linh Nguyen, Carl A Fogarty, Aoife M Harbison, Yves Durocher, John Klassen, Elisa Fadda

**Affiliations:** 1 Department of Chemistry, Maynooth University Maynooth Ireland; 2 Department of Chemistry, University of Alberta Edmonton Canada; 3 Human Health Therapeutics Research Centre, Life Sciences Division, National Research Council Canada Québec Canada; 4 Département de Biochimie et Médecine Moléculaire, Université de Montréal Québec Canada; 5 School of Biological Sciences, University of Southampton Southampton United Kingdom; National Scientific and Technical Research Council Argentina; Iowa State University United States

**Keywords:** SARS-CoV-2, COVID-19, glycosylation, variants, spike, RBD, receptor binding domain, Human

## Abstract

Glycosylation of the SARS-CoV-2 spike (S) protein represents a key target for viral evolution because it affects both viral evasion and fitness. Successful variations in the glycan shield are difficult to achieve though, as protein glycosylation is also critical to folding and structural stability. Within this framework, the identification of glycosylation sites that are structurally dispensable can provide insight into the evolutionary mechanisms of the shield and inform immune surveillance. In this work, we show through over 45 μs of cumulative sampling from conventional and enhanced molecular dynamics (MD) simulations, how the structure of the immunodominant S receptor binding domain (RBD) is regulated by *N*-glycosylation at N343 and how this glycan’s structural role changes from WHu-1, alpha (B.1.1.7), and beta (B.1.351), to the delta (B.1.617.2), and omicron (BA.1 and BA.2.86) variants. More specifically, we find that the amphipathic nature of the *N*-glycan is instrumental to preserve the structural integrity of the RBD hydrophobic core and that loss of glycosylation at N343 triggers a specific and consistent conformational change. We show how this change allosterically regulates the conformation of the receptor binding motif (RBM) in the WHu-1, alpha, and beta RBDs, but not in the delta and omicron variants, due to mutations that reinforce the RBD architecture. In support of these findings, we show that the binding of the RBD to monosialylated ganglioside co-receptors is highly dependent on N343 glycosylation in the WHu-1, but not in the delta RBD, and that affinity changes significantly across VoCs. Ultimately, the molecular and functional insight we provide in this work reinforces our understanding of the role of glycosylation in protein structure and function and it also allows us to identify the structural constraints within which the glycosylation site at N343 can become a hotspot for mutations in the SARS-CoV-2 S glycan shield.

## Introduction

**Figure 1.**
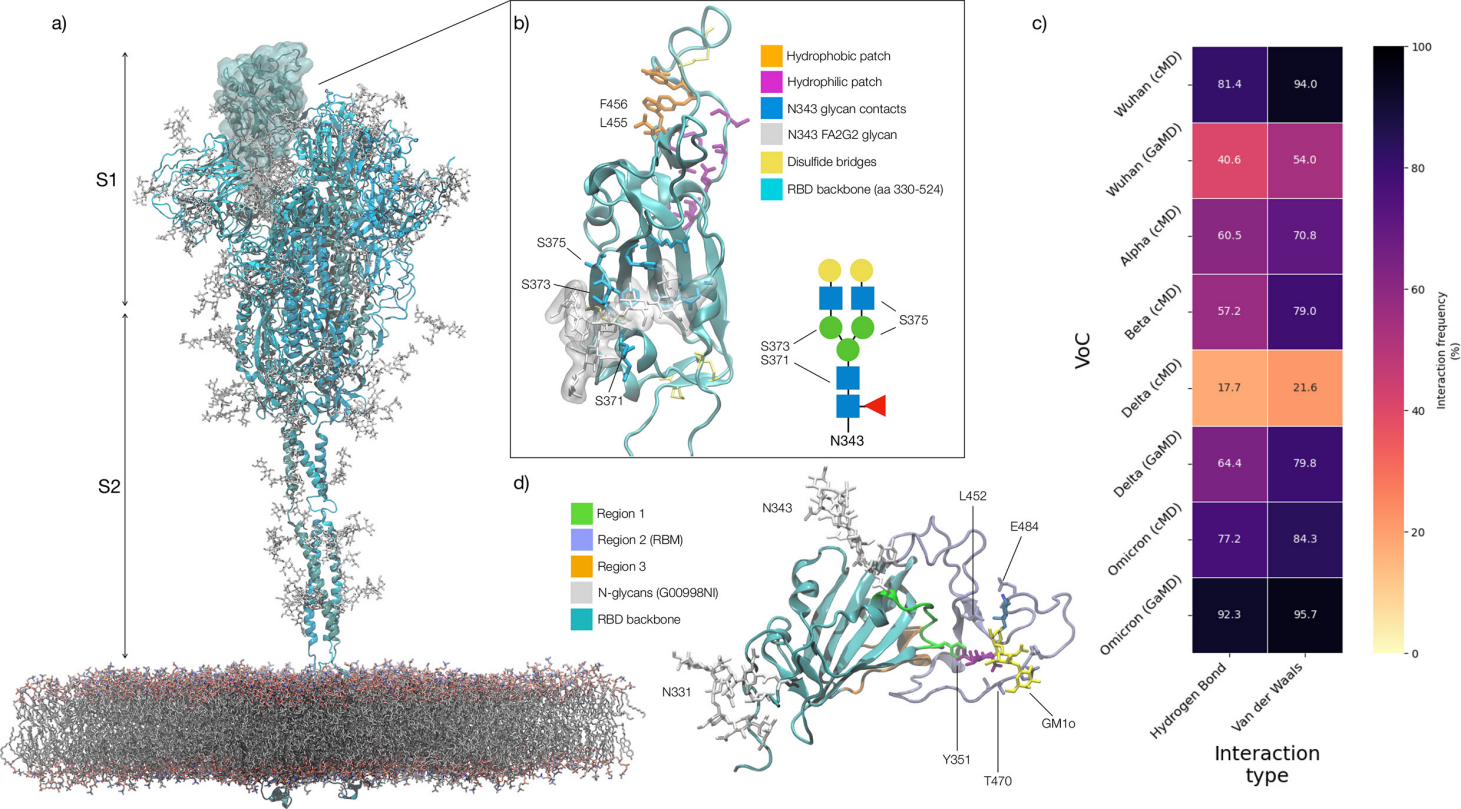
Structure of the SARS-CoV-2 (WHu-1) Spike (S) glycoprotein and of the receptor binding domain (RBD) with a heat map of the interactions between the N343 glycan and the RBD in different variants of concern. (**a**) Atomistic model of the SARS-CoV-2 (WHu-1) S glycoprotein trimer embedded in a lipid bilayer as reported in Casalino et al., 2020. In the conformation shown, the S bears the receptor binding domain (RBD) of chain A in an open conformation, highlighted with a solvent accessible surface rendering. The topological S1 and S2 subdomains are indicated on the left-hand side. Glycans are represented with sticks in white, the protein is represented with cartoon rendering with different shades of cyan to highlight the chains. (**b**) Close-up of the open RBD (WHu-1) in a angiotensin-converting enzyme 2 (ACE2)-bound conformation (PDB 6M0J), with regions colour-coded as described in the legend. Key residues for anchoring the N343 glycan (GlyTouCan-ID G00998NI; FA2G2 Oxford nomenclature), namely S371, S373, and S375, across the beta sheet core are highlighted also in the symbol nomenclature for glycans (SNFG) diagram on the bottom-right with links to the monosaccharides corresponding to primary contacts. Key residues of the hydrophobic patch (orange) found to be inverted in the recently isolated FLip XBB1.5 variant are also indicated. (**c**) Heat map indicating the interactions frequency (%) classified in terms of hydrogen bonding and van der Waals contacts between the N343 glycan and the RBD residues 365–375 for each variants of concern (VoC), over the cumulative conventional MD (cMD) and enhanced gaussian accelerated MD (GaMD) sampling. (**d**) Side view of the RBD highlighting the GM1o binding region (SNFG colouring) and the antigenic Region 1 (green s), Region 2 (or RBM in ice-blue), and Region 3 (orange). Key residues Y351, L452, T470, and E484 are labelled and shown with sticks. N-glycans (white sticks) are labelled according to their linkage to residues Asn 331 and Asn 343. Rendering done with VMD (https://www.ks.uiuc.edu/Research/vmd/).

The SARS-CoV-2 spike (S) glycoprotein is responsible for viral fusion with the host cell, initiating an infection that leads to COVID-19 (Walls et al., 2020; Wrapp et al., 2020). S is a homotrimer with a structure subdivided into two topological domains, namely S1 and S2, see Figure 1a, separated by a furin site, which is cleaved in the pre-fusion architecture (Walls et al., 2020; Wrapp et al., 2020). In the Wuhan-Hu-1 strain (WHu-1), and still in most variants of concern (VoCs), host cell fusion is predominantly triggered by S binding to the angiotensin-converting Enzyme 2 (ACE2) receptor located on the host cell surface (Jackson et al., 2022; Wrapp et al., 2020). This process is supported by glycan co-receptors, such as heparan sulfate (HS) in the extracellular matrix (Clausen et al., 2020; Kearns et al., 2022) and by monosialylated gangliosides oligosaccharides (GM1_os_ and GM2_os_) peeking from the surface of the host cells (Nguyen et al., 2022). The interaction with ACE2 requires a dramatic conformational change of the S, known as ‘opening,’ where one or more receptor binding domains (RBDs) in the S1 subdomain become exposed. The region of the RBD in direct contact with the ACE2 surface is known as receptor binding motif (RBM) (Jackson et al., 2022; Lan et al., 2020; Yi et al., 2020). Ultimately, S binding to ACE2 causes shedding of the S1 subdomain and the transition to a post-fusion conformation, which exposes the fusion peptide near the host cell surface, leading to viral entry (Dodero-Rojas et al., 2021; Jackson et al., 2022).

To exert its functions, S sticks out from the viral envelope where it is exposed to recognition. To evade the host immune system, enveloped viruses hijack the host cell’s glycosylation machinery to cover S with a dense coat of host carbohydrates, known as a glycan shield (Casalino et al., 2020; Chawla et al., 2022; Grant et al., 2020; Turoňová et al., 2020; Watanabe et al., 2020b; Watanabe et al., 2019). In SARS-CoV-2 the glycan shield screens effectively over 60% of the S protein surface (Casalino et al., 2020), leaving the RBD, when open, and regions of the N-terminal domain (NTD) vulnerable to immune recognition (Bangaru et al., 2022; Carabelli et al., 2023; Chawla et al., 2022; Chen et al., 2023; Harvey et al., 2021; Piccoli et al., 2020). The RBD targeted by approximately 90% of serum neutralising antibodies (Piccoli et al., 2020) and thus a highly effective model not only to screen antibody specificity (Du et al., 2020; Lan et al., 2020; Lin et al., 2022) and interactions with host cell co-receptors (Clausen et al., 2020; Mycroft-West et al., 2020; Nguyen et al., 2022), but also as a protein scaffold for COVID-19 vaccines (Dickey et al., 2022; Kleanthous et al., 2021; Montgomerie et al., 2023; Ochoa-Azze et al., 2022; Tai et al., 2020; Valdes-Balbin et al., 2021; Yang et al., 2022).

As a direct consequence, the RBD is under great evolutionary pressure. Mutations of the RBD leading to immune escape are particularly concerning (Cao et al., 2023; Starr et al., 2021), especially when such changes enhance the binding affinity for ACE2 or give access to alternative entry routes (Baggen et al., 2023; Cervantes et al., 2023). The identification of mutational hotspots (Cao et al., 2023) and the effects of mutations in and around the RBM have been and are under a great deal of scrutiny (Starr et al., 2022a; Starr et al., 2022b; Bloom et al., 2023; ; Barton et al., 2021). Yet, less attention is devoted to mutations in the glycan shield, which have been shown to lead to dramatic changes in infectivity (Harbison et al., 2022; Kang et al., 2021; Zhang et al., 2022) and in immune escape (Newby et al., 2023; Pegg et al., 2023). Successful changes in the glycan shield are evolutionarily difficult to achieve, since the nature and pattern of glycosylation of the S is crucial not only to the efficiency of viral entry and evasion, but also to facilitate folding and to preserve the structural integrity of the functional fold. Therefore, identifying potential evolutionary hotspots in the S shield is a complex matter, yet of crucial importance to immune surveillance.

Potential changes of the shield, e.g., loss, shift, or gain of new glycosylation sites, can likely occur only where these do not negatively impact the integrity of the underlying, functional protein architecture. In this work, we present and discuss the case of N343, a key glycosylation site on the RBD. Results of extensive sampling from molecular dynamics (MD) simulations exceeding 45 μs, show how the loss of *N*-glycosylation at N343 affects the structure, dynamics, and co-receptor binding of the RBD, and how these effects are modulated by mutations in the underlying protein, going from the WHu-1 strain through the VoCs designated as alpha (B.1.1.7), beta (B.1.351), delta (B.1.617.2), and omicron (BA.1). In addition, we provide important insight into the structure and dynamics of the omicron BA.2.86 RBD. This variant, designated as variant under monitoring (VUM) and commonly referred to as ‘pirola,’ carries a newly gained *N*-glycosylation site at N354, which represents the first change in the RBD shielding since the ancestral strain.

The SARS-CoV-2 S RBD (aa 327–540) from the WHu-1 (China, 2019) to the EG.5.1 (China, 2023) shows two highly conserved *N*-glycosylation sites (Harbison et al., 2022), one at N331 and the other at N343 (Watanabe et al., 2020a). While glycosylation at N331 is located on a highly flexible region linking the RBD to the NTD, the N343 glycan covers a large portion of the RBD (Casalino et al., 2020; Harbison et al., 2022), stretching across the protein surface and forming a bridge connecting the two helical regions that frame the beta-sheet core, see Figure 1b. In this work, we show that removal of the N343 glycan induces a conformational change which in WHu-1, alpha, and beta allosterically controls the structure and dynamics of the RBM, see Figure 1c. In delta and omicron these effects are significantly dampened by mutations that strengthen the RBD architecture. Further to this molecular insight, we show that enzymatic removal of the N343 glycan affects binding of monosialylated ganglioside co-receptors (Nguyen et al., 2022) in the WHu-1 RBD, but not in delta. We also observe that the affinity of the RBD for GM1 GM1_os_ and GM2 GM1_os_ changes significantly across the VoCs, with beta and omicron exhibiting the weakest binding.

Ultimately, the molecular insight we provide in this work adds to the ever-growing evidence supporting the role of glycosylation in protein folding and structural stability. This information is not only central to structural biology, but also critical to the design of novel COVID-19 vaccines that may or may not carry glycans (Huang et al., 2022), as well as instrumental to our understanding of the evolutionary mechanisms regulating the shield.

## Results

In this section, we start with a brief overview of the architecture of the RBD, we then explain how the RBD structure is modulated by interactions with ACE2 and why the N343 glycan is integral to its stability. We then describe how and why the loss of N343 glycosylation affects the RBD structure and its binding affinity for GM1_os_ and GM2_os_ to different degrees in the VoCs.

### SARS-CoV-2 S RBD structure and antigenicity

The SARS-CoV-2 S RBD encompasses both structured and intrinsically disordered regions. The structured region is supported by a largely hydrophobic beta sheet core, framed by two flanking, partially helical loops (aa 335–345 and aa 365–375), linked by a bridging *N-*glycan at N343, see Figure 1b. The aa 335–345 loop carries the N343 glycosylation site and it is part of an important antigenic region targeted by Class 2 and 3 antibodies (Bangaru et al., 2022; Barnes et al., 2020; Carabelli et al., 2023; Chen et al., 2023). In the bridging conformation, the N343 glycan pentasaccharide extends across the RBD beta sheet to reach the aa 365–375 loop forming highly populated hydrogen bonding and dispersion interactions with the backbone and with the sidechains of residues 365–375, see Figure 1b,c and Appendix 1—figure 2. The bridging N343 glycan shields the hydrophobic beta sheet core of the RBD from the surrounding water, preventing energetically unfavourable contacts. Due to its amphipathic nature, the N343 forms dispersion interactions with the hydrophobic residues of the beta sheet through its core GlcNAc-β(1-4)GlcNAc, while engaging in hydrogen bonds with the surrounding water and with the aa 365–375 helical loop. Notably, the key anchoring residues S371, S373, and S375 within this loop are all mutated to hydrophobic residues in all omicron variants (BA.1–2, BA.4–5, BQ.1.1, EG.5.1, XBB.1.5).

The RBM encompasses aa 439–506 and counts all the RBD residues in direct contact with ACE2 (Lan et al., 2020). The RBM is heavily targeted by both Class 1 and 2 antibodies (Bangaru et al., 2022; Barnes et al., 2020; Carabelli et al., 2023; Chen et al., 2023) and under high evolutionary pressure, with all VoCs carrying mutations in this region. As shown by earlier MD simulations studies (Casalino et al., 2020; Harbison et al., 2022; Sztain et al., 2021; Williams et al., 2022), the RBM in unbound S is largely unstructured and dynamic, an insight also supported by the low resolution cryo-EM maps of this region (Gobeil et al., 2022; Walls et al., 2020; Wrapp et al., 2020). The RBM’s inherent flexibility is likely an important feature in the opening and closing mechanism of the RBD, where the N343 from adjacent RBDs engage with the protein in closed conformation and gate RBD opening (Sztain et al., 2021). The only relatively structured region of the RBM is what we define here on as the hydrophilic patch, see Figure 1b and a hairpin stabilised by a network of interlocking salt-bridges and polar residues, namely R454, R457, K458, K462, E465, D467, S469, and E471, that faces the interior of the S when the RBD is closed.

When in complex with ACE2 or with antibodies, the RBM adopts a structured fold, also shared by the SARS-CoV-1 S RBD (Li et al., 2005). In this conformation, only the terminal hairpin of the RBM (aa 476–486) retains a high degree of flexibility, as shown in this work and by others (Williams et al., 2022). The RBM-bound fold is stabilised by a hydrophobic patch supported by the stacking of the aromatic and aliphatic residues L455, F456, Y473, A475, see Figure 1b, which are part of the protein interface with ACE2. Notably, all residues in the hydrophobic and hydrophilic patches are highly conserved across the VoCs, possibly due to their critical function in inducing and/or stabilising the RBD into its ACE2-bound conformation. As an interesting observation, the loss of stacking in the hydrophobic patch due to the recent F456L mutation in the EG.5.1 variant (China, 2023) is recovered by the L455F mutation in the, appropriately named, FLip variant.

Based on evidence from screenings (Bangaru et al., 2022; Carabelli et al., 2023; Chen et al., 2023), we subdivided the RBD into three different antigenic regions known to be targeted by different classes of antibodies, see Figure 1d. Region 1 stretches from aa 337–353, which includes the N343 glycosylation site, and counts residues targeted by class 2 and 3 antibodies (Bangaru et al., 2022; Carabelli et al., 2023; Harvey et al., 2021). The aa sequence in Region 1 has been highly conserved so far, allowing specific antibodies to retain their neutralisation activity across all VoCs, such as S309 (Newby et al., 2023; Watanabe et al., 2020a), whose binding mode also directly involves the N343 glycan (Liu et al., 2021). A notable and dramatic exception to this high degree of conservation in Region 1 is given by the BA.2.86 variant (Denmark, 2023), known as ‘pirola,’ where the K356T mutation introduces a new *N*-glycosylation sequon at N354. Region 2 coincides with the RBM, which, in addition to binding ACE2 and neutralising antibodies, used to bind the N370 glycan from adjacent RBDs (Allen et al., 2023; Harbison et al., 2022; Watanabe et al., 2020b). N370 glycosylation is lost in SARS-CoV-2 S with the RBM binding cleft available to bind glycan co-receptors, such as glycosaminoglycans (Clausen et al., 2020; Kearns et al., 2022), blood group antigens (Nguyen et al., 2022; Wu et al., 2023), monosialylated gangliosides (Nguyen et al., 2022), among others. Region 3 is a short, relatively structured loop stretching between aa 411–426, located on the opposite side of the RBD relative to Region 1, see Figure 1d.

### Effect of the loss of N343 glycosylation on the structure of the WHu-1, alpha, and beta RBDs

**Figure 2.**
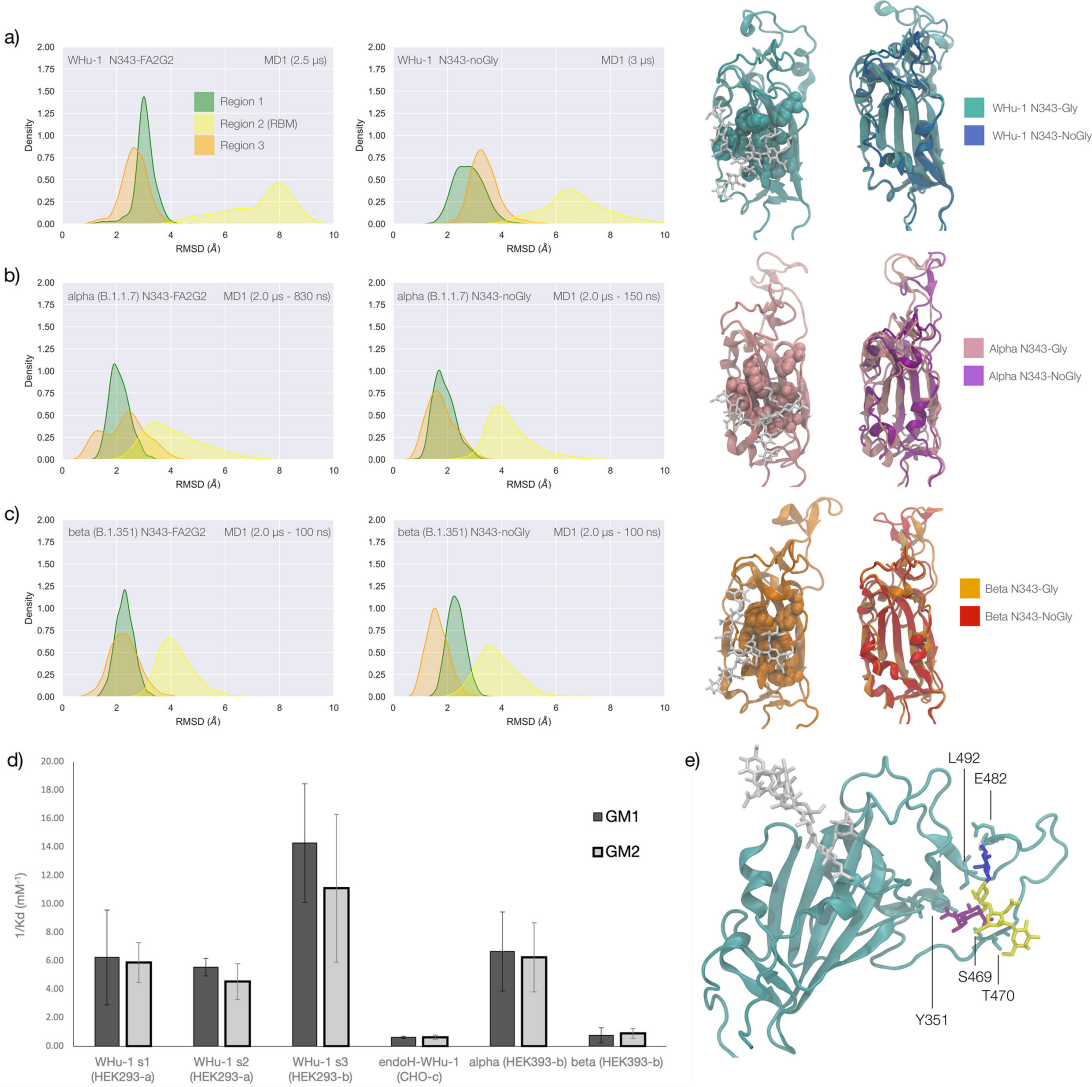
Conformational changes of the S receptor binding domain (RBD) structure in function of loss of N-glycosylation at N343 in WHu-1, alpha and beta variants and binding affinities for interactions between different RBDs and GM1/2os. (**a**) Kernel density estimates (KDE) plot of the backbone RMSD values calculated relative to frame 1 (t=0) of the trajectory for Region 1 (green) aa 337–353, Region 2 (yellow) aa 439–506, and Region 3 (orange) aa 411–426 of the glycosylated (left plot) and non-glycosylated (right plot) WHu-1 RBDs. Duration of the molecular dynamics (MD) sampling is indicated on the top-right corner of each plot with the conformational equilibration time subtracted as the corresponding data were not included in the analysis. Representative structures from the MD trajectories of the WHu-1 RBD glycosylated (cyan) and non-glycosylated (blue) at N343 are shown on the right-hand side of the panel. The N343 glycan (GlyTouCan-ID G00998NI) is rendered with sticks in white, the hydrophobic residues underneath the N343 glycan are highlighted with VDW spheres, while the protein structure is represented with cartoons. (**b**) KDE plot of the backbone RMSD values (see details in (**a**) above) calculated for the alpha (B.1.1.7) RBD glycosylated (left) and non-glycosylated (right) at N343. Representative structures from the MD simulation of the alpha RBDs are shown on the right-hand side of the panel, with the N343 glycosylated RBD shown with pink cartoons and the non-glycosylated alpha RBD in purple cartoons. (**c**) KDE plots of the backbone RMSD values calculated for the beta (B.1.351) RBD glycosylated (left) and non-glycosylated (right) at N343. Representative structures from the MD simulation of the beta RBDs are shown on the right-hand side of the panel, with the N343 glycosylated RBD shown with orange cartoons and the non-glycosylated alpha RBD in red cartoons. (**d**) Binding affinities (1/K_d_, x10^3^ M^–1^) for interactions between different RBDs (including intact and endoF3 treated WHu-1 RBD and alpha and beta RBD) and the GM1_os_ (GlyTouCan-ID G46613JI) and GM2_os_ (GlyTouCan-ID G61168WC) oligosaccharides. HEK293a samples (Nguyen et al., 2022) and shown here as reference. HEK293b samples all carry FLAG and His tags and are shown for WHu-1 (glycosylated and treated with endoF3 treated), alpha, and beta sequences. Further details are in Appendix 1. (**e**) Predicted complex between the WHu-1 RBD and GM1_os_, with GM1_os_ represented with sticks in symbol nomenclature for glycans (SNFG) colours, the protein represented with cartoons (cyan), and the N343 with sticks (white). Residues directly involved in the GM1_os_ binding or proximal are labelled and highlighted with sticks. All N343 glycosylated RBDs carry also a FA2G2 N-glycan (GlyTouCan-ID G00998NI) at N331, which is not shown for clarity. Rendering done with VMD (https://www.ks.uiuc.edu/Research/vmd/), KDE analysis with seaborn (https://seaborn.pydata.org/), and bar plot with MS Excel.

Results obtained for the WHu-1 strain and for the alpha (B.1.1.7) and beta (B.1.351) VoCs are discussed together due to their sequence and structure similarity, with alpha counting only one mutation (N501Y) and beta three mutations (K417N, E484K, and N501Y) relative to the WHu-1 RBD. Extensive sampling through conventional MD, i.e., 4 μs for the alpha and beta VoC and 8 μs with an additional 4 μs of Gaussian accelerated MD (GaMD) for the WHu-1 RBD, see Appendix 1—table 1, shows that the loss of N343 glycosylation induces a dramatic conformational change in the RBD, where one or both helical loops flanking the hydrophobic beta sheet core pull towards each other, see Figure 2. This conformational change can occur very rapidly upon removal of the *N*-glycan or after a longer delay due to the complexity of the conformational energy landscape. The data used for the analysis corresponds to systems that have reached structural stability, i.e., equilibrium; we discarded the timeframes corresponding to conformational transitions.

To explore the effects of the loss of N343 glycosylation in the WHu-1 RBD, we started the MD simulations from different conformations. In one set of conventional sampling MD trajectories (MD1) and in the GaMD simulations the starting structure corresponds to an open RBD from an MD equilibrated S ectodomain obtained in earlier work (Harbison et al., 2022). In this system, the RBM is unfolded and retains the maximum degree of flexibility. MD2 was started from a conformation corresponding to the ACE2-bound structure (Lan et al., 2020). Results obtained from MD2 and GaMD are entirely consistent with results from MD1, and thus are included as Appendix 1—figure 1. The GaMD simulation shows a lower degree of contact of the N343 glycan with the aa 365–375 stretch of the opposite loop, see Figure 1c, because most of the contacts are with residues further downstream from position 365. Nevertheless, the N343 remains engaged in a bridging conformation throughout the simulation. As shown by the RMSD values distributions, represented through Kernel Density Estimates (KDE) in Figure 2, the structure of Region 1 in the WHu-1 RBD is stable. In the glycosylated RBD, the stability of Region 1 is largely due to the contribution of the bridging N343 glycan, chosen here to be a complex biantennary FA2G2 form based on Watanabe et al., 2020a, forming hydrogen bonds with the residues in loop aa 365–375 throughout the simulations, see Figure 1c and Appendix 1—figure 2. Conversely, the conformation of the RBM (Region 2) is very flexible in both glycosylated and non-glycosylated forms. Loss of N343 glycosylation triggers a conformational change in the Region 1 of the WHu-1 RBD, shown by a broader KDE peak in Figure 2a. This conformational change ultimately triggers the complete detachment of the hydrophilic loop from Region 1, see Figure 1c, through rupture of the non-covalent interactions network between Y351 (Region 1) and S469 or T470 (Region 2) via of hydrogen bonding, and Y351 and L452 (Region 2) via CH-π stacking. Structural changes in Region 3 upon loss of glycosylation at N343 appear to be negligible.

The starting structure used for the simulations of the alpha RBD derives from the ACE2-bound conformation of the WHu-1 RBD (PDB 6M0J) modified with the N501Y mutation. The reconstructed glycan at N343 interacts with the aa 365–375 throughout the entire trajectory, but it adopts a stable conformation only after 830 ns, where we started collecting the data shown in Figure 2b. Again, we see that the loss of glycosylation at N343 causes a swift conformational change that brings the aa 335–345 and aa 365–375 loops closer together, see Figure 2b. This conformational change involves primarily Region 1, and just like the previous case, it ultimately determines the detachment of the hydrophilic patch from the Y351 in Region 1. Also shown by the KDE plot in Figure 2b and a small conformational change in Region 3, which involves a partial disruption and refolding of a helical turn, can be observed during the trajectory of the N343 glycosylated alpha RBD. As in the previous case, the structure of Region 3 appears to be unaffected by N343 glycosylation, at least within the sampling accumulated in this work.

In the beta RBD (starting structure from PDB 7LYN) the reconstructed N343 glycan adopts a bridging conformation quite rapidly and retains this conformation throughout the trajectory with only minor deviations. The corresponding RMSD values KDE distributions for Regions 1–3, see Figure 2c, reflect this structural stability. The stability of the RBM (Region 2) is supported by interactions between Y351 (Region 1) and the hydrophilic loop, as noted earlier. Loss of glycosylation at N343 causes a rapid tightening of the RBD core helical loops towards each other, which again in this case ultimately causes the detachment of the hydrophilic loop from Y351 in Region 1 towards the end of the MD trajectory, i.e., after 1.9 μs of sampling.

### Effect of the loss of N343 glycosylation on the binding affinity of GM1_os_ /2 _os_ for the WHu-1, alpha, and beta RBDs

Earlier work from some of us shows that the WHu-1 RBD binds monosialylated gangliosides as co-receptors in SARS-CoV-2 infection (Nguyen et al., 2022). The 3D structure of the low affinity complex between the GM1_os_ and the WHu-1 RBD has been proven difficult to obtain experimentally, yet we were able to obtain a promising model through a combination of multiple structural alignments of the GM1os to the WHu-1 RBD-bound N370 (Harbison et al., 2022) and extensive MD simulations, details in Appendix 1 and in Newby et al., 2023; Watanabe et al., 2020a. The predicted GM1_os_ binding site is located at the junction between Region 1 and Region 2 of the WHu-1 RBD and it involves all the residues that stabilise the region, namely Y351, L452, S469, and T470, see Figures 1c and 2e. As part of our investigation of glycan co-receptors binding to the SARS-CoV-2 RBD, we used direct ESI-MS assay to determine the impact of the loss of N343 glycosylation on GM1_os_ and GM2_os_ binding. Here, we used endoF3-treatment to trim down the fucosylated biantennary and triantennary complex *N*-glycans into core nonfucosylated or fucosylated GlcNAc (Gn or GnF, respectively). LC-MS analysis suggests that *N*-glycans on N343 but not N331 of WHu-1 RBD were trimmed down (Appendix 1—figure 6). From the zero-charge mass spectra of endoF3-treated WT RBD (Appendix 1—figure 5), we performed glycan assignment (Appendix 1—table 8) and found that 31% of detected glycoforms contained Gn/GnF at N343, while the remaining was the intact form. Affinity data in Figure 2d show that the enzymatic removal of the N343 glycan from the WHu-1 RBD causes a complete loss of GM1_os_/2_os_ binding, which is consistent with both, the involvement of the junction between Regions 1 and 2 the binding and its allosteric control of the RBM dynamics. Furthermore, while binding of GM1_os_ and GM2_os_ to the alpha RBD appears to be slightly decreased relative to WHu-1, binding to the beta RBD is dramatically reduced. We can reconcile this finding with the mutation E484K in beta, which changes the key interaction between E484 and GM1_os_, see Figure 2 and with changes in structure and dynamics of the RBM terminal hairpin induced by mutations (Williams et al., 2022), which have also been suggested to affect the S opening kinetics (Wang et al., 2021).

### Effects of N343 glycosylation on the structure of the delta RBD

**Figure 3.**
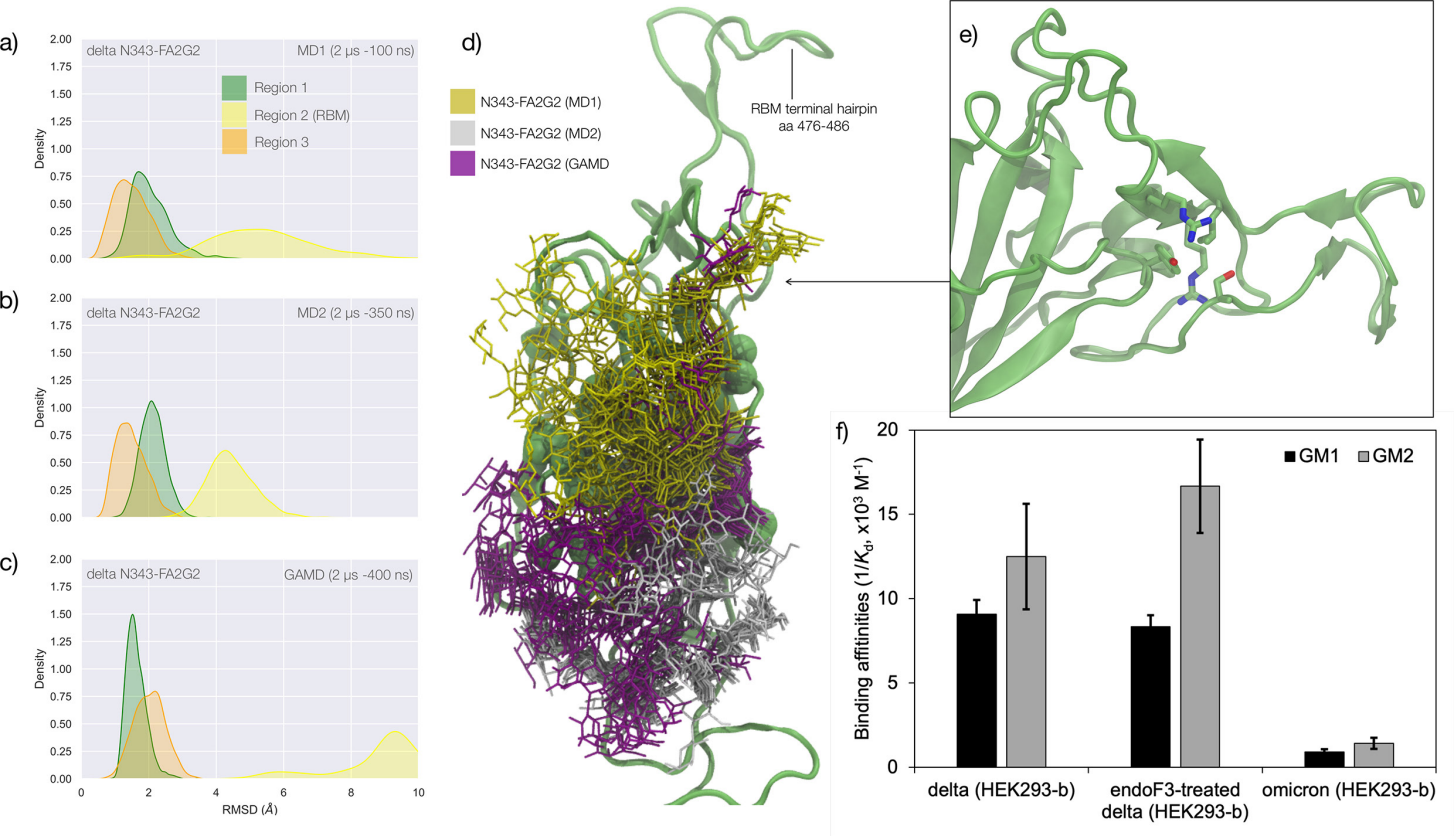
Conformational analysis and 3D structure of the S receptor binding domain (RBD) in the delta variant glycosylated at N343 with a diagram showing binding affinity to GM1/2os in the presence and absence of the glycan at N343 and in the omicron variant. (**a**) Kernel density estimates (KDE) plot of the backbone RMSD values calculated relative to frame 1 (t=0) of the MD trajectories (MD1) trajectory for Region 1 (green) aa 337–353, Region 2 (yellow) aa 439–506, and Region 3 (orange) aa 411–426 of the N343 glycosylated delta (B.1.617.2) RBD. The MD1 simulation was started from the open RBD conformation from the cryo-EM structure PDB 7V7Q. Based on the conformation of the N-glycan reconstructed at N353, the first 100 ns of the MD1 production trajectory were considered part of the conformational equilibration and not included in the data analysis. (**b**) KDE plot of the backbone RMSD values calculated relative to frame 1 (t=0) of the MD2 trajectory for Regions 1–3 (see details above) of the N343 glycosylated delta (B.1.617.2) RBD. The MD2 simulation was started from the open RBD conformation from the cryo-EM structure PDB 7V7Q with different velocities relative to MD1. The first 350 ns of the MD2 production trajectory were considered part of the conformational equilibration and not included in the data analysis. (**c**) KDE plot of the backbone RMSD values calculated relative to frame 1 (t=0) of the gaussian accelerated MD (GaMD) trajectory for Regions 1–3 of the N343 glycosylated delta (B.1.617.2) RBD. The first 400 ns of the GaMD production trajectory were considered part of the conformational equilibration and not included in the data analysis. (**e**) Graphical representation of the delta RBD with the protein structure (lime cartoon) from a representative snapshot from MD1. The N343 FA2G2 glycan (GlyTouCan-ID G00998NI) is represented in different colours, corresponding to the different molecular dynamics (MD) trajectories, as described in the legend, with snapshots taken at intervals of 100 ns. Residues in the hydrophobic core of the delta RBD are represented with VDW spheres partially visible under the *N*-glycans overlay. (**f**) Insert showing the junction between Regions 1 and 2 from the left-hand side of the RBD in (**e**). The residues involved in the network solidifying the junction are highlighted with sticks and labelled. (**f**) Affinities (1/K_d_, x10^3^ M^–1^) for interactions between GM1_os_ (GlyTouCan-ID G46613JI) and GM2_os_ (GlyTouCan-ID G61168WC) oligosaccharides and the intact and endoF3-treated delta RBD and omicron RBD. Rendering done with VMD (https://www.ks.uiuc.edu/Research/vmd/), KDE analysis with seaborn (https://seaborn.pydata.org/), and bar plot with MS Excel.

The delta (B.1.617.2) RBD carries two mutations, namely L452R and T478K, relative to the WHu-1 strain. The open RBD in the cryo-EM structure PDB 7V7Q was used as starting conformation for the MD simulations of both the glycosylated and the non-glycosylated delta RBDs. To understand how the mutations in delta affect the RBD structure and modulate the response to the loss of glycosylation at N343, we ran two uncorrelated conventional MD simulations (2 μs) and one GaMD simulation (2 μs) for both the glycosylated and non-glycosylated systems, for a total (cumulative) sampling of 12 μs. Results are shown in Figure 3. In the glycosylated delta RBD the N343 glycan is observed to be much more dynamic than in the WHu-1, alpha, and beta RBDs, engaging in contacts with different regions of the RBD in addition to the loop aa 365–375. In response to these fluctuations, the conformation of the RBD remains stable with only minor deviations from the average structure of Regions 1 and 3. All trajectories, and in particular the results of the GaMD simulation in Figure 3c, show that RBM (Region 2) of delta is highly dynamic. This flexibility appears to involve specifically the terminal hairpin of the RBM (aa 476–486), which includes the T478K mutation, while the rest of the RBM is tightly anchored due to the L454R mutation. More specifically, the R452 of delta can establish a new hydrogen bond with Y351, in addition to S469 and T470, reinforcing the junction between Regions 1 and 2. Furthermore, in the delta RBM the role of L452 in CH-π stacking to Y351 is taken by the proximal L492, through a twist of the beta sheet, see Figure 3e. These interactions also contribute to reinforcing the R454 orientation, tightening the link with the RBM hydrophilic patch.

The effect of the loss of glycosylation at N343 on the delta RBD was assessed by running two uncorrelated MD simulations, one by conventional sampling (MD1 of 3 μs), and the other through enhanced sampling (GaMD of 2 μs). As a consequence of the L452R mutation shown in Figure 3, the tightening of the helical loops aa 335–345 and aa 365–375 over the hydrophobic core of the RBD occurring upon loss of glycosylation at N343 does not affect the structure and dynamic of the junction between Regions 1 and 2, see Appendix 1—figure 3. Results of the conventional MD simulation show that the tightening of the loops is mainly achieved by a larger displacement of the aa 365–375 loop rather than of Region 1, while the GaMD results show tightening of both loops, see Appendix 1—figure 3. In all simulations, the structure of the junction between Regions 1 and 2 remains undisturbed, with no detachment of the hydrophilic patch within the sampling we collected.

### Effect of the loss of N343 glycosylation on the binding affinity of GM1/2 for the delta RBD

To examine the effect of N343 glycosylation on glycan binding of delta RBD, we used the direct ESI-MS assay to quantify the binding affinities between endo F3-treated delta and GM1_os_ and GM2_os_. From the zero-charge mass spectra of endoF3-treated RBD, see Appendix 1—figure 5, we performed glycan assignment, see Appendix 1—table 9, and found that both N331 and N343 glycans were trimmed down to Gn/GnF. Direct ESI-MS data in Figure 3f show no loss of GM1/2 binding in the delta RBD upon loss of N343 glycosylation, which further supports the involvement of the Region 1–2 junction in sialylated glycans recognition.

### Effects of N343 glycosylation on the RBD structure in the omicron BA.1 SARS-CoV-2

**Figure 4.**
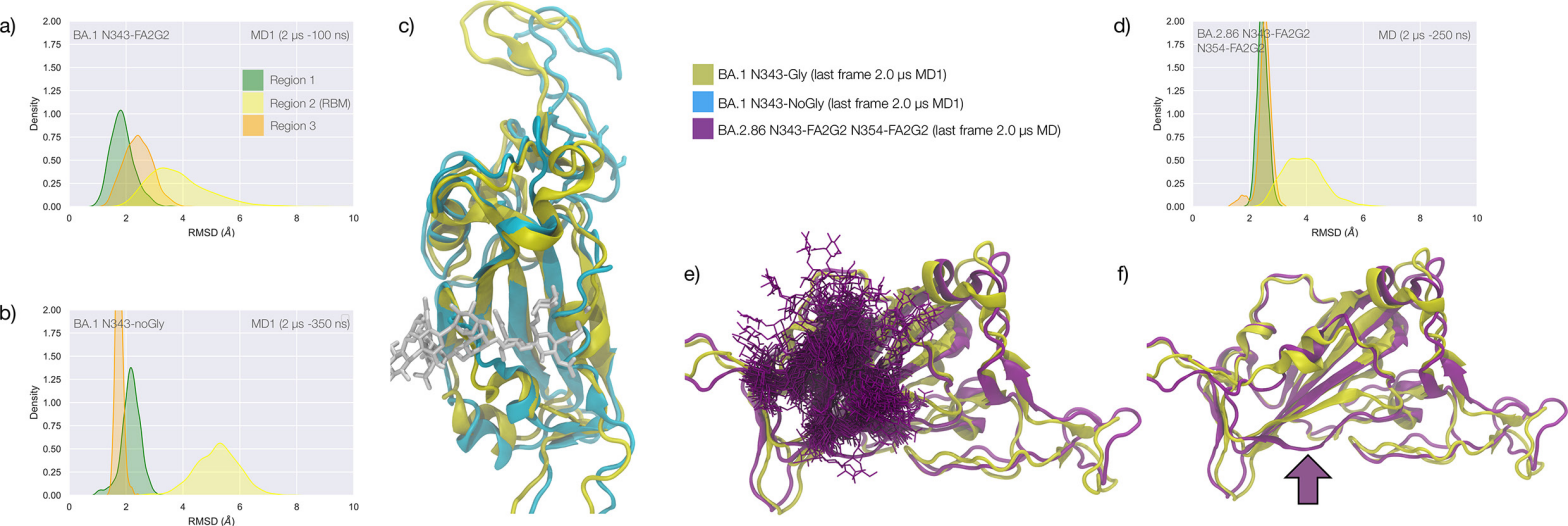
Conformational dynamics of the BA.1 and BA.2.86 S receptor binding domain (RBD) in function of N343 glycosylation. (**a**) Kernel density estimates (KDE) plot of the backbone RMSD values calculated relative to frame 1 (t=0) of the gaussian accelerated MD (GaMD) trajectory for Region 1 (green) aa 337–353, Region 2 (yellow) aa 439–506, and Region 3 (orange) aa 411–426 of the glycosylated omicron (BA.1) RBD. (**b**) KDE plot of the backbone RMSD values calculated relative to frame 1 (t=0) of the GaMD trajectory (see details above) of the non-glycosylated omicron (BA.1) RBD. (**c**) Graphical representation of the glycosylated (protein in yellow cartoons and N343-FA2G2 in white sticks, N331 omitted for clarity) and non-glycosylated (protein in cyan cartoons) of the omicron (BA.1) RBD. Structures correspond to the last frame of the GaMD trajectories, see details in the legend. (**d**) KDE plot of the backbone RMSD values calculated relative to frame 1 (t=0) of the molecular dynamics (MD) trajectory of the omicron BA.2.86 RBD glycosylated with FA2G2 N-glycans at N343, N354 and N331 (not shown). (**e**) Graphical representation of the omicron BA.2.86 RBD (protein in violet cartoons and N-glycans in violet sticks) structurally aligned to the glycosylated omicron (BA.1) RBD (protein in yellow cartoons) for reference. The N343 and N354 glycans are intertwined throughout the trajectory. (**f**) Same graphical representation of the omicron BA.2.86 and BA.1 RBDs with the N-glycans not shown. The purple arrow points to the displacement of the loop in response to the presence of the N354 glycan in BA.2.86. Rendering with VMD (https://www.ks.uiuc.edu/Research/vmd/) and KDE analysis with seaborn (https://seaborn.pydata.org/).

The omicron BA.1 RBD carries 15 mutations relative to the WHu-1 strain, namely S371L, S373P, S375F, K417N, N440K, G446S, S477N, T478K, E484A, Q493R, G496S, Q498R, N501Y, Y505H, and T547K. The S371L, S373P, and S375F mutations, retained in all omicron VoCs including the most recently circulating XBB.1.5, EG.5.1, and BA.2.86, remove all hydroxyl sidechains that we have seen being involved in hydrogen bonding interactions with the N343 glycan in the WHu-1, alpha, beta, and delta RBDs, see Figure 1c and Appendix 1—figure 3. We investigated the effects of the loss of glycosylation at N343 in the structure of the BA.1 RBD through two sets of uncorrelated conventional MD simulations (MD1 and MD2) and one set of GaMD, with total cumulative simulation time of 12 μs. Starting structures correspond to the open RBD in PDB 7QO7 (MD1) and in PDB 7WVN (MD2 and GaMD), where the N343 glycan was reconstructed in different conformations, depending on the spatial orientation of the N343 sidechain. The results of the MD1 and GaMD simulations show that, despite the S371L, S373P, and S375F mutations, the N343 glycan is still forms stable contacts with the aa 365–375 loop, see Figure 1c and Appendix 1—figure 3**,** and these interactions contribute to the stability of the RBD structure, see Figure 4. In the starting structure, we used for MD2 the N343 glycan was built with the core pentasaccharide pointing away from the RBD hydrophobic core. Consequently, the *N*-glycan adopts different transient conformations during the MD2 trajectory, which terminate with an interaction with the hydrophobic interior of the RBD and with the N331 glycan, see Appendix 1—figure 6. In all simulations, the loss of glycosylation at N343 causes a tightening of the aa 335–345 and aa 365–375 loops, which in omicron is stabilised by more efficient packing of the aa 365–375 loop within the hydrophobic core, driven by the embedding of the L371 and F375 sidechains. The non-glycosylated RBD adopts a stable conformation where we do not see a detachment of the hydrophilic patch. The stabilising effect of the aa 365–375 loop mutations in omicron could not be tested by means of affinity for GM1_os_/2_os_ as omicron (BA.1) binds those epitopes only weakly, see Figure 3f. Based on the binding site we predicted by MD simulations, see Figures 1d and 2e, and as observed for beta, the loss of E484 due to the E484A mutation in omicron may negate GM1_os_/2_os_ binding.

The stability of the RBD structure is further enhanced by the presence of an additional glycosylation site at N354, which appeared in the recently detected omicron BA.2.86 ‘pirola’ variant. As shown in Figure 4d–f, the *N-*glycans at N343 and N354 are tightly intertwined throughout the trajectory stabilising Region 1, also shielding the area very effectively. The presence of an additional *N-*glycosylation site at N354 also changes the conformation of the loop that hosts the site relative to the BA.1 starting structure we used as a template to run the MD simulation, see Figure 4f. To note, based on earlier glycoproteomics analysis (Newby et al., 2023; Watanabe et al., 2020a) and on the exposure to the solvent of the reconstructed glycan structure at N354, we chose to occupy all glycosylation sites with FA2G2 *N*-glycans.

## Discussion

Quantifying the role of glycosylation in protein folding and structural stability is a complex task due to the dynamic nature of the glycan structures (Fadda, 2022; Woods, 2018) and to the micro- and macro-heterogeneity in their protein functionalization (Čaval et al., 2021; Riley et al., 2019; Struwe and Robinson, 2019; Thaysen-Andersen and Packer, 2012; Zacchi and Schulz, 2016) that hinder characterization. Yet, the fact that protein folding occurs within a context where glycosylation types and occupancy can change on the fly, suggests that not all glycosylation sites are essential for the protein to achieve and retain a native fold and that those sites may be displaced without consequences to function. In this work, we investigated the structural role of the *N-*glycosylation at N343 in the SARS-CoV-2 S RBD, one of the most highly conserved sites in the viral phylogeny (Harbison et al., 2022). Extensive MD simulations in this and in earlier work by us and others (Casalino et al., 2020; Grant et al., 2020; Harbison et al., 2022; Sikora et al., 2021) show that the RBD core is efficiently shielded by this glycan. Furthermore, the N343 glycan has been shown to be mechanistically involved in the opening and closing of the S (Sztain et al., 2021), making this glycosylation site functionally essential towards viral infection.

In this work, we performed over 45 μs of cumulative MD sampling with both conventional and enhanced schemes to show that the N343 glycan also plays a fundamental structural role in the WHu-1 SARS-CoV-2 and that this role has changed in the variants circulating thus far. While we cannot gauge how fundamental is N343 glycosylation towards correct folding, we see that the amphipathic nature of the complex *N*-glycan (Watanabe et al., 2020a) at N343 enhances the stability of the RBD architecture, bridging between the two partially helical loops that frame a highly hydrophobic beta sheet core. To note, we determined the same bridging structures also for oligomannose types *N*-glycans at N343 in earlier work (Harbison et al., 2022). In all variants, we observe that the removal of the glycan at N343 triggers a tightening of the loops in a response likely aimed at limiting access of water into the hydrophobic core. In WHu-1, alpha, and beta RBDs this event allosterically controls the dynamics of the RBM, ultimately causing the detachment of the hydrophilic patch and misfolding from the ACE2-recognized conformation. These results are in agreement with the drastic reduction of viral infectivity observed upon deletion of both N331 and N343 glycosylation in the WHu-1 strain (Li et al., 2020), where loss of structure may add to the loss of function through gating (Sztain et al., 2021) or vice versa.

As a functional assay to support this molecular insight, we determined how the binding affinity of the RBD for the oligosaccharides of the monosialylated gangliosides GM1_os_ and GM2_os_ is modulated by N343 glycosylation. These were shown in earlier work by us and others to function as co-receptors in WHu-1 infection (Nguyen et al., 2022). We predicted through extensive MD sampling that GM1_os_ and GM2_os_ bind the RBD into a site corresponding precisely to the location occupied by the 6-arm of an ancestral *N*-glycan at N370 (Garozzo et al., 2022; Harbison et al., 2022). Note, that the N370 site is still occupied by zoonotic sabercoviruses (Allen et al., 2023). The GM1_os_ binding site, see Figure 2e, is located precisely at the junction between Regions 1 and 2, which is disrupted by the loss of N343 glycosylation in WHu-1. Accordingly, we find that enzymatic removal of the N343 glycan abolishes GM1_os_ and GM2_os_ binding in the WHu-1 RBD, see Figure 2d. While we expect a similar loss of binding in alpha, within the context of a lower affinity relative to the WHu-1 RBD, we find that the beta RBD does not bind GM1_os_ and GM2_os_, regardless of its glycosylation state. Based on the structure of the GM1-RBD complex we identified, see Figure 2e, where E484 represents a key contact to the oligosaccharides, the mutation of E484K in beta may be key to the loss of binding, together with change in the RBM kinetics linked to this mutation and to variations within the same region (Wang et al., 2021; Williams et al., 2022).

In the delta variant, we observed that the L452R mutation is responsible for an increased structural stability of the RBD, reinforcing the non-covalent interactions network between Region 1 and the RBM. Indeed, the tightening of the loops occurring upon loss of N343 glycosylation does not trigger a misfolding of the RBM, see Figure 3. Accordingly, we observe that the delta RBD with the trimming down of N331 and N343 glycans shows no significant change in binding affinity for GM1_os_ and GM2_os_ relative to the fully glycosylated form, see Figure 3f.

In all omicron variants, including all the currently circulating VoCs and VUMs, the loop aa 365–375 that the N343 glycan hooks on, carries similar mutations, with the highly conserved S371, S373, and S375 all mutated to hydrophobic residues, see Appendix 1—figure 1. Our MD results on the BA.1 and BA.2.86 RBDs show that hydrophobic residues at positions 371, 372, and 373 can pack within the RBD core, while leading to a loop structure that can support the N343 glycan branches through interactions with the backbone, see Figure 4. We have shown for all variants that the contacts between the N343 glycan and the aa 365–375 stretch of the opposite loop are fairly equally distributed, between hydrophilic (hydrogen bonding) and hydrophobic (dispersion or van der Waals) type interactions, see Figure 1c. Therefore, it is expected that the loss of anchoring hydrogen bonding residues can be supported through other interactions. Within this context, the removal of the N343 glycan does still cause a tightening of the loops, yet through a different mechanism relative to the other variants that ultimately does not appear to affect the RBM dynamics. As in beta, for omicron, there is negligible binding of the N343 glycosylated RBD to GM1_os_ and GM2_os_, likely due to the E484A mutation, which based on the predicted structure of the GM1os/RBD complex, see Figures 1d and 2e, would deny a key interaction within the predicted binding site.

Taken together, our results show that since the WHu-1, alpha, and beta strains, the RBD has evolved to make the *N-*glycosylation site at N343 structurally dispensable. Within this framework, provided that an *N*-glycosylation site in the immediate vicinity of N343 is necessary for folding and for function, a shift of the site within the sequence can potentially occur. Such a modification may negatively affect recognition and binding by neutralising antibodies (Liu et al., 2021; Piccoli et al., 2020; Pinto et al., 2020) and thus promote evasion. We have also shown for the BA.2.86 that the new glycosylation site at N354 can effectively contribute to the stability of Region 1, while significantly increasing shielding.

Moreover, we show that specific VoCs lost affinity for monosialylated ganglioside oligosaccharides with a trend in agreement with a binding site located at the junction between Region 1 and the RBM, which is part of the N370 glycan binding cleft on the RBD (Harbison et al., 2022). This conclusion is further supported by how binding affinities for GM1_os_ and GM2_os_ change upon the loss of N343 glycosylation, in agreement with the MD results. Further to this, as mutations we identified dampened binding to monosialylated ganglioside oligosaccharides, it is also possible that further mutations may switch the affinity back on or determine a shift of preference of the RBD towards other glycans that can still be recognised within the N370 cleft. Further work is ongoing in this area.

Finally, the results from this work point to the importance of understanding the impact of *N-*glycosylation on protein structure and stability, with immediate consequences to COVID-19 vaccine design. Indeed, earlier work shows SARS-CoV-2 S-based protein vaccines with increased efficacy due to the removal of *N*-glycans (Huang et al., 2022), and of RBD-based vaccines in use and under development (Cohen et al., 2022; Más-Bermejo et al., 2022; Valdes-Balbin et al., 2021) that may be designed with and without *N-*glycans. The design of such constructs may benefit from understanding which *N-*glycosylation sites are structurally essential and which are dispensable. Further to this, our results show that taking into consideration the effects on *N-*glycosylation on protein structural stability and dynamics in the context of specific protein sequences, may be key to understanding epistatic interactions among RBD residues (Rochman et al., 2022; Witte et al., 2023), which would be otherwise difficult, where not impossible, to decipher.

## Materials and methods

### Computational methods

All simulations were performed using additive, all-atom force fields, namely the AMBER 14 SB parameter set (Maier et al., 2015) to represent protein atoms and counterions (200 mM of NaCl), GLYCAM06j-1 (Kirschner et al., 2008) to represent glycans, and TIP3P for water molecules (Jorgensen et al., 1983). All production trajectories from conventional (deterministic) MD simulations were run for a minimum of 2 μs to ensure convergence. In some cases, we extended the simulations up to 3 μs to assess the stability of specific conformational transitions, where deemed necessary. All Gaussian accelerated MD (GaMD) (Miao et al., 2015; Wang et al., 2021) production trajectories were run for 2 μs. All simulations of the N343 glycosylated and non-glycosylated RBDs were started from identical 3D structures. The glycans at N331 and N343 were rebuilt as FA2G2 (GlyTouCan-ID G00998NI) based on glycoproteomics data (Newby et al., 2023; Watanabe et al., 2020a) with 3D structures from our GlycoShape database (Ives et al., 2023) (https://glycoshape.org). Further information on the RBD structures and PDB IDs for all variants, together with details on the MD systems set-up, equilibration protocols, and total sampling times allocations are available in Appendix 1. Sequences for all VoCs and VUM RBDs (aa 327–540) from https://viralzone.expasy.org/9556.

### Proteins and glycans

Expression and purification of recombinant WHu-1, Alpha, Beta, Delta, and Omicron RBD (EG^319^RVQP…VN^541^F, UniProt number P0DTC2) with C-terminal FLAG (SGDYKDDDDKG) and His tags (HHHHHHG) used in the current study were described elsewhere (Akache et al., 2021; Colwill et al., 2022). Mutations of SARS-CoV-2 RBD VOCs are shown in Appendix 1—figure 1. Proteins were purified using standard immobilised metal-ion affinity chromatography (IMAC), followed by size-exclusion chromatography on Superdex-75 to remove dimers as described (Forest-Nault et al., 2022). To obtain endo F3-treated WHu-1 and Delta RBD, 100 μg of each RBD was treated with endo F3 (purchased from New England Biolabs) in 1 x Glycobuffer (50 mM sodium acetate, pH 4.5) at 37 °C overnight. Each protein was dialyzed and concentrated against 100 mM ammonium acetate (pH 7.4) using an Amicon 0.5 mL microconcentrator (EMD Millipore) with a 10 kDa MW cutoff and stored at –80 °C until used. The concentrations of protein stock solutions were estimated by UV absorption (280 nM). The oligosaccharides of GM1 and GM2, Galβ1-3GalNAcβ1-4(Neu5Acα2–3)Galβ1-4Glc (MW 998.34 Da, GM1_os_), and GalNAcβ1-4(Neu5Acα2–3)Galβ1-4Glc (MW 836.29 Da, GM2_os_), respectively, were purchased from Elicityl SA (Crolles, France). 1 mM stock solutions of each glycan were prepared by dissolving a known mass of glycan in ultrafiltered Milli-Q water. All stock solutions were stored at –20 °C until needed.

### ESI-MS affinity measurements

Affinities (*K*_d_) of glycan ligands for RBD were measured by the direct ESI-MS binding assay. The ESI-MS affinity measurements were performed in positive ion mode on a Q Exactive Orbitrap mass spectrometer (Thermo Fisher Scientific). The capillary temperature was 150 °C, and the S-lens RF level was 100; an automatic gain control target of 5 × 10^5^ and a maximum injection time of 100 ms were used. The resolving power was 17,500. The instrument was equipped with a modified nanoflow ESI (nanoESI) source. NanoESI tips with an outer diameter (o.d.) of ∼5 µm were pulled from borosilicate glass (1.2 mm o.d., 0.69 mm i.d., 10 cm length, Sutter Instruments, CA) with a P-97 micropipette puller (Sutter Instruments). A platinum wire was inserted into the nanoESI tip, making contact with the sample solution, and a voltage of 0.8 kV was applied. Each sample solution contained a given RBD (5 μM) and GM1_os_ or GM2_os_ (at three different concentrations ranging from 10 to 150 μM) in ammonium acetate (100 mM, pH 7.4). Data acquisition and pre-processing was performed using the Xcalibur software (version 4.1); ion abundances were extracted using the in-house software SWARM (Kitov et al., 2019). A brief description of the data analysis procedures used in this work is given as the Supporting Information.

### Protease digestion

20 µg of a given purified protein (intact and endoF3-treated WT RBDs) were dissolved in 100 μL of 8 M urea in 100 mM Tris-HCl (pH 8.0) containing 3 mM EDTA and incubated at room temperature for 1 hr. The denatured protein was then reduced with 5 μL of 500 mM dithiothreitol (DTT; Sigma-Aldrich) at room temperature for 1 hr; followed by alkylation with 12 µL of 500 mM iodoacetamide (Sigma-Aldrich) at room temperature for 20 min in the dark. The reaction was quenched by adding 5 µL of 250 mM DTT, and the solution buffer was exchanged using a 10 kDa Amicon Ultra centrifugal filter. The samples were loaded onto the filter and centrifuged at 14,000 × g for 15 min. The glycoprotein solution was subsequently digested with trypsin/chymotrypsin (substrate/enzyme (wt/wt) = 50) in 50 mM ammonium bicarbonate (pH 8.0) for 18 hr at 37 °C. The reaction was quenched by heat inactivation at 100 °C for 10 min. The lyophilized sample was stored at –20 °C until LC-MS analysis.

### Peptide analysis by reverse-phase liquid chromatography (RPLC)-MS/MS

The digested samples were separated using a RPLC-MS/MS on a Vanquish UHPLC system (Thermo Fisher Scientific) coupled with ESI-MS detector (Thermo Q Exactive Orbitrap). Peptide separation was achieved using a Waters Acquity UPLC Peptide BEH C18 column (1.7 μm, 2.1 mm × 150 mm; Waters). The eluents were 0.1% formic acid in water (solvent A) and 0.1% formic acid in acetonitrile (solvent B). The separation was performed at 60 °C. The following gradient was used for MS detection: t = 0 min, 95% solvent A (0.2 mL min^–1^); t = 45 min, 40% solvent A (0.2 mL min^–1^); t = 55 min, 5% solvent A (0.2 mL min^–1^); t = 55.1 min, 95% solvent A (0.2 mL min^–1^). During LC-MS analysis, the following parameters were used: sheath gas flow rate of 10 arbitrary units (AU), capillary temperature of 250 °C, and spray voltage of 1.5 kV. The mass spectra were acquired in positive mode with an m/z range of 200–3000 at a resolution of 70,000. The automatic gain control target was set at 1 × 10^6^, and a maximum injection time of 100ms was used. HCD mass spectra were acquired in the data-dependent mode for the five most abundant ions with a resolution of 17,500. Automatic gain control target, maximum injection time, and isolation window were set at 2 × 10^5^, 200 ms, and 2.0 m/z, respectively. HCD-normalized collision energy was 25%. The data were recorded by Xcalibur (Thermo, version 4.1) and analyzed using Thermo BioPharma Finder software.

The peptide sequences (EG^319^RVQP…VN^541^FS with C-terminal FLAG (SGDYKDDDDKG) and His tags (HHHHHHG), UniProt number P0DTC2) were then identified using the theoretical digest feature of the software. Carbamidomethylation and carboxymethylation at cysteine residues were used as a fixed modification. Common mammalian *N*- and *O*-glycans were also used as variable modifications. A precursor mass tolerance of 5 ppm was set. For quantification, the abundance of each *N*-glycan at each *N*-glycosylation site (N331 and N343) is the sum of MS areas under the peak curve divided by the corresponding charge states. Next, for each *N*-glycosylation site, the relative abundance of each *N*-glycan is calculated as its abundance over the total abundance of all *N*-glycans detected.

## Figures and Tables

**Appendix 1—figure 1.**
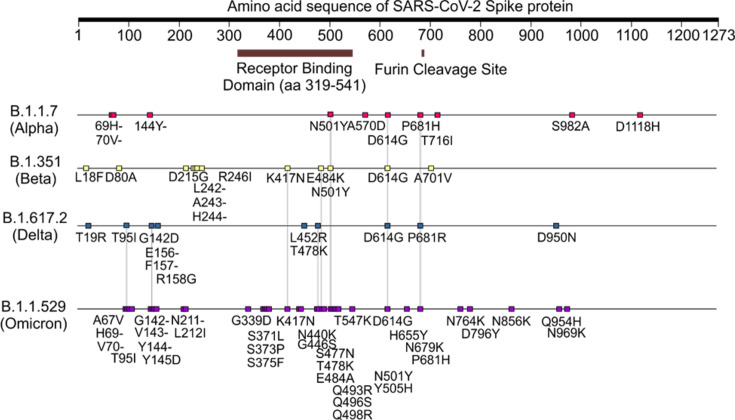
Amino acid sequence of SARS-CoV-2 spike (S) protein variants and mutations on receptor binding domain (RBD) used in this study.

**Appendix 1—figure 2.**
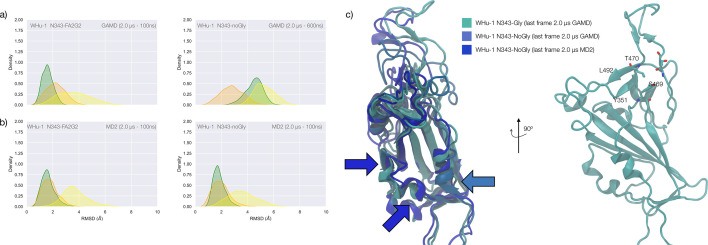
Conformational dynamics of the WHu-1 receptor binding domain (RBD) in function of N343 glycosylation. (**a**) Kernel density estimates (KDE) plots of the backbone RMSD values calculated relative to frame 1 (t=0) of the gaussian accelerated MD (GaMD) trajectory for Region 1 (green) aa 337–353, Region 2 (yellow) aa 439–506, and Region 3 (orange) aa 411–426 of the N343 glycosylated and non-glycosylated WHu-1 RBD. The GaMD simulations were started from the structure of the RBD in PDB 6M0J. The first 100 ns of the N343 glycosylated RBD trajectory were considered part of the conformational equilibration and not included in the data analysis. The first 600 ns of the trajectory obtained for the N343 non-glycosylated WHu-1 RBD were considered part of the conformational equilibration and not included in the data analysis. (**b**) KDE plots of the backbone RMSD values calculated relative to frame 1 (t=0) of the conventional molecular dynamics (MD) trajectory MD2 (see details above). MD2 was started from the conformation of the RBD in PDB 6M0J. The first 100 ns of both trajectories were considered part of the conformational equilibration and not included in the data analysis. (**c**) Graphical representation of the structural alignment of N343 glycosylated and non-glycosylated RBDs from the last frames of the GaMD and MD2 trajectories. Colour-coded arrows (see legend) indicate where the main conformational changes leading to the tightening of the helices occur in each system. Proteins are represented by cartoons and N343 glycan is not represented for clarity. On the right-hand side a rotation of the RBD by 90°Clockwise shows how the hydrophilic loop in Region 2 is still well connected to Y351 in Region 1 at the end of the GaMD N343 glycosylated WHu-1 RBD. Rendering done with VMD (https://www.ks.uiuc.edu/Research/vmd/) and KDE analysis with seaborn (https://seaborn.pydata.org/).

**Appendix 1—figure 3.**
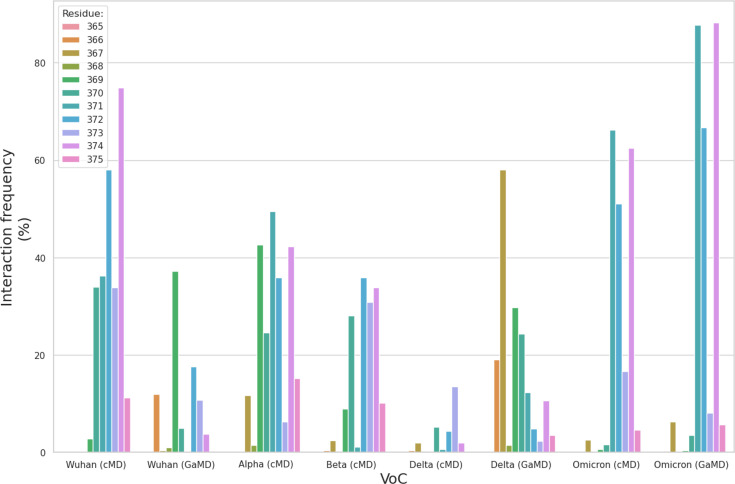
Bar plot of the interaction frequencies (%) of the N343 *N-*glycan with the different residues within the aa 365–375 loop for each variants of concern (VoC). The interactions include both hydrogen bonding and dispersion (van der Waals) contacts.

**Appendix 1—figure 4.**
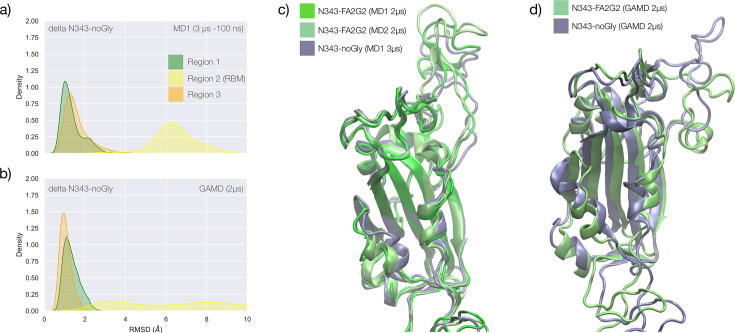
Conformational dynamics of the delta receptor binding domain (RBD) in function of N343 glycosylation. (**a**) Kernel density estimates (KDE) plot of the backbone RMSD values calculated relative to frame 1 (t=0) of the MD1 trajectory for Region 1 (green) aa 337–353, Region 2 (yellow) aa 439–506, and Region 3 (orange) aa 411–426 of the N343 non-glycosylated delta RBD. (**a**) KDE plot of the backbone RMSD values calculated relative to frame 1 (t=0) of the gaussian accelerated MD (GaMD) trajectory of the N343 non-glycosylated delta RBD. (**c**) Graphical representations of the delta RBD structures from the last frame of the conventional simulation MD1 (N343 glycosylated and non-glycosylated) and MD2 (N343 glycosylated) with colourings indicated in the legend. Protein is represented with cartoons and the N343 and N331 glycans are omitted for clarity. (**c**) Graphical representations of the structurally aligned delta RBD structures from the last frame of the accelerated simulation GaMD (N343 glycosylated and non-glycosylated). Protein is represented with cartoons and the N343 and N331 glycans are omitted for clarity. Rendering done with VMD (https://www.ks.uiuc.edu/Research/vmd/) and KDE analysis with seaborn (https://seaborn.pydata.org/).

**Appendix 1—figure 5.**
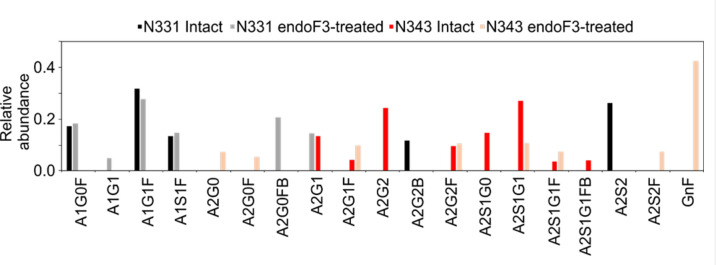
Relative abundance of *N*-glycans at N331 and N343 on the wild-type (WT) receptor binding domain (RBD) before and after endoF3 treatment.

**Appendix 1—figure 6.**
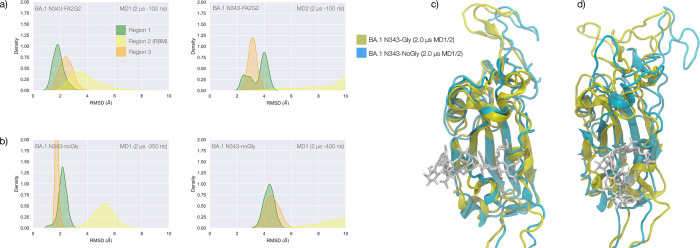
Conformational dynamics of the BA.1 receptor binding domain (RBD) in function of N343 glycosylation. (**a**) Kernel density estimates (KDE) plot of the backbone RMSD values calculated relative to frame 1 (t=0) of the MD1 (left) and MD2 (right) trajectories for Region 1 (green) aa 337–353, Region 2 (yellow) aa 439–506, and Region 3 (orange) aa 411–426 of the glycosylated omicron (BA.1) RBD. (**b**) KDE plot of the backbone RMSD values calculated relative to frame 1 (t=0) of the MD1 and MD2 trajectories (see details above) of the non-glycosylated omicron (BA.1) RBD. (**c**) Graphical representation of the structural alignment of the glycosylated (protein in yellow cartoons and N343-FA2G2 in white sticks, N331 omitted for clarity) and non-glycosylated (protein in cyan cartoons) of the omicron (BA.1) RBD from MD1. (**d**) Graphical representation of the structural alignment of the glycosylated (protein in yellow cartoons and N343-FA2G2 in white sticks, N331 omitted for clarity) and non-glycosylated (protein in cyan cartoons) of the omicron (BA.1) RBD from MD2. Structures correspond to the last frames of the trajectories, see details in the legend. Rendering done with VMD (https://www.ks.uiuc.edu/Research/vmd/) and KDE analysis with seaborn (https://seaborn.pydata.org/).

## Data Availability

All MD simulations data are available open access on https://doi.org/10.5281/zenodo.10441732. The following dataset was generated: FaddaE 2024MD simulations from "#GotGlycans: Role of N343 Glycosylation on the SARS-CoV-2 S RBD Structure and Co-Receptor Binding Across Variants of ConcernZenodo10.5281/zenodo.10441732PMC1116874438864493

## Additional files

MDAR checklist

## Appendix 1

### Materials and methods

#### MD simulations

The RBD simulation systems were constructed from residues R327 to N540 of the SARS-CoV-2 S glycoprotein. The starting structure for WHu-1 MD1 was from the open RBD obtained from the simulation of the S ectodomain (Casalino et al., 2020), and the WHu-1 MD2 starting structure from PDB 6M0J. The alpha (B.1.1.7) starting structure was obtained from PDB 6M0J, with the N501Y mutation introduced with the mutagenesis tool in pymol (https://pymol.org/2/). The beta starting structures were from PDB 7LYN. All delta starting structures (MD1 and MD2, glycosylated and non-glycosylated) were obtained from PDB 7V7Q, with MD1 and MD2 productions started from different velocities. The omicron starting structures for MD1 was from PDB 7WVN, and the starting structure for MD2 from PDB 7QO7. Simulations of BA.2.86 were performed from PDB 7WVN, with sequence mutations introduced with the mutagenesis tool in pymol.

The charged N- and C-terminal residues were neutralised by capping with acetyl (ACE) and N-methylamide (NME) groups, respectively. The RBD was glycosylated with FA2G2 glycans at N331 and at N343. The structure of the (GlyTouCan-ID G00998NI) N-glycan was sourced from the GlycoShape glycan structure database (GDB), and was linked to the RBD using the Re-Glyco, a glycoprotein builder tool developed for GlycoShape (https://glycoshape.org). The systems were solvated in a water box with a minimum distance of 12 Å, and ions were added to neutralise any system charges to a total concentration of 200 mM NaCl.

All simulations were performed using AMBER18 (Case et al., 2018) on resources provided by the Irish Centre for High-End Computing (ICHEC). The AMBER 14 SB force field (Maier et al., 2015) was used to model proteins and ions, the GLYCAM06j-1 (Kirschner et al., 2008) force field was used to model glycans, and the TIP3P water model was used to model solvent molecules (Jorgensen et al., 1983).

The energy of the system was minimised in 500,000 steps using the steepest descent algorithm, with all heavy atoms of the protein and glycan restrained with a potential weight of 5 kcal.mol^–1^.Å^–2^. The system was then equilibrated in the NVT ensemble, with the system gradually heated from 0 to 100 K, and then from 100 K to 300 K. The system was then equilibrated in the NPT ensemble to maintain the pressure at 1 bar. Following this, most restraints were then removed, with restraints of a magnitude of 5 kcal.mol^–1^.Å^–2^ remaining in place on the caps, R327-N334, and G526-N540. This is because these regions of the RBD would be connected to the other domains of the spoke glycoprotein, and so would be structurally constrained in vivo. The system was then equilibrated for a further 100 ns, before production runs of ~2 μs, as summarised in Appendix 1—table 1.

The temperature was maintained at 300 K using Langevin dynamics with a collision frequency of 1 ps^–1^, and the pressure was maintained at 1 bar using isotropic position scaling with a Berendsen barostat and a pressure relaxation time of 2 ps. Periodic boundary conditions were used throughout the simulations. The Van der Waals interactions were truncated at 11 Å and Particle Mesh Ewald (PME) was used to treat long-range electrostatics with B-spline interpolation of order 4. The SHAKE algorithm was used to constrain all bonds containing hydrogen atoms and to allow the use of a 2 fs time step for all simulations.

**Appendix 1—table 1.** Molecular dynamics (MD) sampling methods and corresponding times in the production phase of the simulations. MD1 and MD2 indicate trajectories collected through conventional (deterministic) sampling. Gaussian accelerated MD (GaMD) indicates trajectories obtained through Gaussian accelerated MD sampling. Further details on the methodology and starting structures are in the text.

	N343-FA2G2 (μs)			N343 NoGly (μs)		
Variant	MD1	MD2	GaMD	MD1	MD2	GaMD
WHu-1	2.5	2.0	2.0	3.0	2.0	2.0
Alpha (B.1.1.7)	2.0	-	-	-	2.0	-
Beta (B.1.351)	2.0	-	-	-	2.0	-
Delta (B.1.617.2)	2.0	2.0	2.0	3.0	-	2.0
Omicron (BA.1)	2.0	2.0	2.0	2.0	2.0	2.0
Omicron (BA.2.86)	2.0	-	-	-	-	-
Total (46.5)	12.5	6.0	6.0	8.0	8.0	6.0

Additionally, Gaussian-accelerated MD (GaMD) simulations (Miao et al., 2015; Wang et al., 2021) were run using the GaMD module in AMBER18 (Case et al., 2018). GaMD simulations were performed for glycosylated and non-glycosylated RBD systems of the Whu-1, delta, and omicron variants. For these GaMD simulations, the starting structure for the Whu-1 variant was obtained from the simulation of the S ectodomain (Casalino et al., 2020), and the starting structures for the delta and omicron variants from PDBs 7V7Q and 7WVN, respectively. In brief, a 2 ns short conventional MD simulation was used to collect the required potential statistics for calculating GaMD acceleration parameters. This was followed by a 50 ns equilibration after adding the boost potential, and finally a 2 μs GaMD production simulation. The average and standard deviation of the system potential energies were calculated every 0.4 ns. GaMD simulations were run at the ‘dual-boost’ level by setting the reference energy to the lower bound. One boost potential was applied to the dihedral energetic term, and the other to the total potential energetic term. The upper limit of the boost potential standard deviation was set to 6 kcal.mol^–1^ for both the dihedral and total potential energetic terms. The same temperature and pressure parameters were used in the conventional MD simulations.

#### Structure of the RBD-GM1o Complex

The equilibrium structure of the unbound RBD was obtained from MD simulations of the SARS-CoV-2 spike (S) WHu-1 ectodomain (Harbison et al., 2022) and was screened for binding to the GM1 tetrasaccharide (GM1o) (Nguyen et al., 2022). The GM1o structure was built with the GLYCAM Carbohydrate Builder and equilibrated by MD simulations in bulk water separately from the RBD. The same GM1o structure is deposited in the GlycoShape GDB (https://glycoshape.org) where it can be downloaded. As docking did not produce any convincing binding poses, none of which proved to be stable when tested by MD simulations, we used the conformation of the N370 N-glycan bound across the RBD (Harbison et al., 2022) as a guideline for the generation of a set of potential RBD/GM1o complexes. The stability of all promising conformations produced this way was tested by extensive sampling through MD simulations, started with a restrained equilibration phase (100–500 ns), where the protein was equilibrated around the bound (fixed) GM1o, followed by unrestrained MD. The complex shown in Figure 1d was obtained after numerous binding and unbinding events that occurred during the unrestrained MD, and it remained stable for 700 ns. As an interesting note, the position of the Neu5Ac in the bound GM1o corresponds to the position that a terminal sialic acid in N370 would have when bound to the RBD.

#### Assignment of glycan compositions of RBD glycoforms

For each RBD VOCs, the data were analysed using the measured molecular weights (MWs) of intact protonated WT, Alpha, Beta, Delta, and Omicron RBD (WTx, Ax, Bx, Dx, and Ox respectively, Appendix 1—tables 2–6). The MW of deglycosylated RBD was calculated based on the elemental composition corresponding to amino acid sequence (EG^319^RVQP…VN^541^FS, UniProt number P0DTC2) and any amino acid substitutions plus FLAG tag (SGDYKDDDDKG) and hexa histidine tag (HHHHHHG) and four disulfide bonds. The MWs of non-glycosylated RBD WT, Alpha, Beta, Delta, and Omicron are 27,352.6 Da, 27,401.6 Da, 27,386.6 Da, 27,422.7 Da, and 27,613.9 Da, respectively. Possible glycan compositions were simulated for the numbers of N-acetylhexosamines (N: HexNAc, N-acetylgalactosamine, and N-acetylglucosamine), hexoses (H: Hex, glucose, and galactose), fucoses (F: Fuc), and N-acetylneuraminic acids (S: Neu5Ac). Possible values of N, H, F, and S were calculated by considering reported *N*- and *O*-glycans. Possible MWs of RBD glycoforms were then calculated from the sum of aforementioned MW of RBD and MWs of glycan residues from each possible H_N_F_S combination.

#### ESI-MS affinity measurements

The affinities of glycan ligands for RBD were measured by the direct ESI-MS binding assay (Kitova et al., 2012). For a monovalent protein-ligand (PL) interaction (Equation S1), the dissociation constant (*K*_d_; Equation S2) can be calculated from the ratio (*R*) of total abundances of L-bound (*Ab*(PL)) to free P ions (*Ab*(P), Equation S3) measured by ESI-MS.

(S1) P+L⇄PL

(S2) $$K_{d}=\frac{\left[P][L\right]}{\left[PL\right]}=\frac{{\left[L\right]}_{0}}{R}-\frac{{\left[P\right]}_{0}}{R+1}$$

(S3) $$R=\frac{Ab(PL)}{Ab(P)}=\frac{\left[PL\right]}{\left[P\right]}$$

where [P]_0_ and [L]_0_ are initial concentrations of P and L, respectively. The abundance ratio *R* measured by ESI-MS is taken to be equal to the equilibrium concentration ratio in the solution. Because RBD consists of multiple species with distinct glycan compositions, and glycosylation can, in principle, influence binding, the affinities for L binding to individual RBD species (Equation S4a and Equation S4b) were determined.

(S4a) $$P_{1}+L⇄P_{1}L$$

(S4b) $$P_{x}+L\;\;P_{x}L$$

The corresponding equations of mass balance equations are,

(S5a) $${\left[L\right]}_{0}=\left[L\right]+\sum_{x}\left[P_{x}L\right]$$

(S5b) $$\left[P_{1}{]}_{0}=[P_{1}]+[P_{1}L\right]$$

(S5c) $$\left[P_{2}{]}_{0}=[P_{2}]+[P_{2}L\right]$$

(S5d) $$\left[P_{x}{]}_{0}=[P_{x}]=[P_{x}L\right]$$

where [P_x_]_0_ is the initial concentration of a given P_x_ species. Initial concentrations of individual RBD species were estimated from their relative abundances measured by ESI-MS, assuming uniform response factors (Equation S6).

(S6) $${\left[{\text{P}}_{\text{x}}\right]}_{\text{0}}=\frac{Ab\left({\text{P}}_{\text{x}}\right)}{\sum_{x}Ab\left({\text{P}}_{x}\right)}{\left[\text{P}\right]}_{\text{0}}$$

The affinity of a given P_x_ species (*K*_dx_) was calculated from Equation S7

(S7) $${\text{K}}_{dx}=\frac{\left[P_{x}\right]\left[L\right]}{\left[P_{x}L\right]}=\frac{{\left[L\right]}_{0}}{{\text{R}}_{\text{x}}}-\frac{1}{{\text{R}}_{\text{x}}}\sum_{x}\frac{{\text{R}}_{\text{x}}{\left[P_{x}\right]}_{0}}{\left(1+{\text{R}}_{\text{x}}\right)}$$

where *R_x_* is the total abundance ratio of ligand-bound and free P*_x_* ions (Equation S8).

(S8) $${\text{R}}_{\text{x}}=\frac{\text{Ab}\left(P_{x}L\right)}{\text{Ab}\left(P_{x}\right)}=\frac{{\left[P_{x}L\right]}_{eq}}{{\left[P_{x}\right]}_{eq}}$$

In some instances, signal for one ligand-bound P_x_ complex overlapped with signal for another (free) P_x_ species. Spectral overlap was corrected for by considering the abundance ratio of the two P_x_ species (for example P_x_ and P_x+1_) in the absence of L (*r*; Equation S9) and assuming that P_x_ and P_x+1_ exhibit identical affinities for L.

(S9) $$r=\frac{Ab\left(P_{x+1}\right)}{Ab\left(P_{x}\right)}$$

The corresponding *r* value was then used to calculate the true *R*_x_ value (*R_x,corr_*; Equation S10) corrected for spectral overlap,

(S10) $$R_{x,cor}=\frac{Ab\left(P_{x}L\right)}{Ab\left(P_{x}\right)}-r$$

which was used in Equation S7 to calculate *K*_dx_.

**Appendix 1—table 2.** Affinities of the oligosaccharides of GM1 and GM2 (GM1_os_ and GM2_os_, respectively) for WHu-1, Alpha, Beta, Delta, and Omicron receptor binding domain (RBD) and endoH-treated WHu-1 RBD and endoF3-treated WHu-1 and Delta RBD measured by ESI-MS for aqueous ammonium acetate (100 mM, pH 7.4) solutions containing a given RBD (5 μM) and glycan (three different initial concentrations ranging from 10 μM to 150 μM). Data represent mean ± SD; n = 3 independent experiments for each glycan concentration.

Variant	GM1_os_ *K*_d_ (mM)	GM2_os_ *K*_d_ (mM)
WHu-1 RBD (sample 1; HEK293-a)	0.16 ± 0.04	0.17 ± 0.02
WHu-1 RBD (sample 2; HEK293-a)	0.18 ± 0.01	0.22 ± 0.03
WHu-1 RBD (sample 3; HEK293-b)	0.07 ± 0.01	0.09 ± 0.02
endoF3-treated WHu-1 RBD (HEK293-b)	3.6 ± 0.7	5.7 ± 0.6
alpha (HEK293-b)	0.15 ± 0.03	0.16 ± 0.03
beta (HEK293-b)	1.3 ± 0.4	1.10 ± 0.2
delta (HEK293-b)	0.11 ± 0.01	0.08 ± 0.02
endoF3-treated delta (HEK293-b)	0.12 ± 0.01	0.06 ± 0.01
micron (HEK293-b)	1.1 ± 0.2	0.70 ± 0.16

**Appendix 1—table 3.** Summary of molecular weights (MWs) of WHu-1 receptor binding domain (RBD) glycoforms (WTx) with respective relative abundances identified by ESI-MS and putative H_N_F_S combinations.

RBD	MeasuredMW (Da)	Relative abundance	H_N_F_S combination	Theoretical MW (Da)	Mass difference (Da)
WT1	31342.4	25.5	9_11_2_0	31345.1	5.3
WT2	31402.4	2.6	11_9_1_1	31408.0	2.4
WT3	31505.2	42.7	9_9_0_3	31520.1	6.9
WT4	31527.0	34.3	10_9_1_2	31537.1	2.1
WT5	31609.4	19.0	11_10_3_0	31612.1	5.3
WT6	31668.8	48.8	10_9_0_3	31682.1	5.4
WT7	31690.2	56.2	11_9_1_2	31699.1	1.0
WT8	31711.4	17.8	9_10_0_3	31723.2	3.7
WT9	31771.1	43.9	12_10_3_0	31774.2	4.9
WT10	31794.0	65.6	10_11_0_2	31797.2	4.8
WT11	31816.5	26.5	10_9_1_3	31828.2	3.7
WT12	31835.3	30.6	11_9_2_2	31845.2	1.9
WT13	31854.4	44.6	9_10_1_3	31869.2	6.8
WT14	31875.2	47.5	10_10_2_2	31886.2	3.1
WT15	31896.4	35.8	11_10_1_2	31902.2	2.2
WT16	31935.8	5.6	10_11_1_2	31943.3	0.6
WT17	31957.8	85.0	11_11_0_2	31959.2	6.6
WT18	31979.7	98.1	13_11_0_1	31992.3	4.6
WT19	31001.2	61.3	9_10_2_3	32015.3	6.1
WT20	32038.7	17.3	11_10_0_3	32047.3	0.6
WT21	32060.1	55.7	12_10_1_2	32064.3	3.9
WT22	32083.3	55.9	10_11_0_3	32088.3	3.0
WT23	32105.5	1.1	12_11_0_2	32121.3	7.8
WT24	32125.1	12.6	13_11_1_1	32138.3	5.2
WT25	32144.3	72.6	10_12_1_2	32146.3	5.9
WT26	32165.0	63.5	12_12_1_1	32179.3	6.4
WT27	32185.7	39.8	11_10_1_3	32193.3	0.4
WT28	32247.8	88.4	11_11_0_3	32250.3	5.5
WT29	32270.3	100.0	13_11_0_2	32283.4	5.1
WT30	32291.8	62.4	12_14_1_0	32294.4	5.4
WT31	32348.8	58.7	12_10_1_3	32355.4	1.4
WT32	32371.5	46.6	10_11_0_4	32379.4	0.1
WT33	32434.4	69.7	10_12_1_3	32437.4	5.0
WT34	32456.1	76.4	12_12_1_2	32470.4	6.3
WT35	32477.0	43.6	11_10_1_4	32484.4	0.6
WT36	32538.2	78.0	11_11_0_4	32541.4	4.8
WT37	32560.9	79.6	13_11_0_3	32574.4	5.6
WT38	32583.0	49.5	12_14_1_1	32585.5	5.5
WT39	32615.2	19.1	11_10_0_5	32629.5	6.3
WT40	32637.9	60.5	12_10_1_4	32646.5	0.6
WT41	32659.6	39.0	10_11_0_5	32670.5	2.9
WT42	32725.4	55.2	10_12_1_4	32728.5	4.9
WT43	32747.7	58.2	12_12_1_3	32761.5	5.8
WT44	32768.8	4.1	11_10_1_5	32775.5	1.3
WT45	32826.8	1.7	11_11_0_5	32832.5	2.3
WT46	32851.5	10.7	13_11_0_4	32865.5	6.1
WT47	32873.7	4.3	12_14_1_2	32876.6	5.1
WT48	32927.5	49.5	12_10_1_5	32937.6	2.1
WT49	32949.8	15.6	10_11_0_6	32961.6	3.8
WT50	33293.5	34.6	13_11_1_5	33302.7	1.2
WT51	33583.8	9.4	13_11_1_6	33593.8	2.0

**Appendix 1—table 4.** Summary of molecular weights (MWs) of B.1.1.7 receptor binding domain (RBD) glycoforms (Alpha, Ax) with respective relative abundances identified by ESI-MS and putative H_N_F_S combinations.

RBD	MeasuredMW (Da)	Relative abundance	H_N_F_S combination	Theoretical MW (Da)	Mass difference (Da)
A1	30894.6	6.4	9_10_0_0	30899.0	4.3
A2	31080.7	17.1	9_8_2_1	31076.0	4.7
A3	31137.8	5.3	9_9_3_0	31134.1	3.7
A4	31160.2	3.2	9_7_2_2	31164.0	3.8
A5	31184.9	3.7	9_10_0_1	31190.1	5.2
A6	31243.1	13.0	10_8_2_1	31238.1	5.1
A7	31265.9	10.9	10_11_0_0	31264.1	1.8
A8	31300.5	4.5	10_9_3_0	31296.1	4.4
A9	31319.6	3.4	8_10_2_1	31320.1	0.5
A10	31346.6	2.6	10_10_0_1	31352.1	5.5
A11	31369.5	19.4	11_10_1_0	31369.1	0.4
A12	31405.1	26.4	8_9_2_2	31408.1	3.0
A13	31427.3	13.6	11_11_0_0	31426.1	1.1
A14	31449.4	4.9	7_10_4_1	31450.2	0.8
A15	31482.0	4.6	9_10_2_1	31482.2	0.1
A16	31505.7	3.4	7_11_3_1	31507.2	1.5
A17	31529.1	30.9	12_10_1_0	31531.2	2.0
A18	31553.5	35.3	10_11_0_1	31555.2	1.7
A19	31590.8	27.8	12_11_0_0	31588.2	2.6
A20	31611.6	16.5	8_10_2_2	31611.2	0.4
A21	31658.9	9.8	11_10_3_0	31661.2	2.3
A22	31693.9	8.8	13_10_1_0	31693.2	0.6
A23	31715.2	96.9	11_11_0_1	31717.2	2.0
A24	31737.6	13.7	12_11_1_0	31734.3	3.3
A25	31773.3	19.8	9_10_2_2	31773.3	0.1
A26	31793.9	13.4	12_12_0_0	31791.3	2.7
A27	31819.9	31.4	12_10_1_1	31822.3	2.4
A28	31844.4	20.4	10_11_0_2	31846.3	1.9
A29	31880.1	13.3	12_11_0_1	31879.3	0.8
A30	31901.1	78.6	8_10_4_2	31903.3	2.3
A31	31920.8	4.8	11_12_0_1	31920.3	0.5
A32	31937.3	2.7	12_12_1_0	31937.3	0.1
A33	31957.6	10.8	13_12_0_0	31953.3	4.2
A34	31978.1	10.0	9_11_2_2	31976.3	1.7
A35	31006.1	100.0	11_11_0_2	31008.3	2.2
A36	32027.7	5.2	12_11_3_0	32026.4	1.3
A37	32061.8	4.9	9_10_2_3	32064.4	2.6
A38	32081.4	53.5	12_12_0_1	32082.4	1.0
A39	32109.6	10.5	12_10_1_2	32113.4	3.8
A40	32137.5	13.8	10_11_2_2	32138.4	0.9
A41	32191.8	82.8	13_11_3_0	32188.4	3.4
A42	32212.7	3.2	11_12_0_2	32211.4	1.3
A43	32227.5	2.6	12_12_1_1	32228.4	1.0
A44	32266.5	39.1	9_11_2_3	32267.4	0.9
A45	32297.9	48.2	11_11_0_3	32299.4	1.5
A46	32372.0	53.5	12_12_0_2	32373.5	1.5
A47	32393.9	5.0	11_10_4_2	32389.5	4.4
A48	32446.4	31.2	11_11_1_3	32445.5	0.9
A49	32483.1	35.2	13_11_3_1	32479.5	3.6
A50	32504.3	4.7	11_12_2_2	32503.5	0.7
A51	32557.2	43.9	14_12_3_0	32553.6	3.7
A52	32590.0	10.0	11_11_2_3	32591.5	1.6
A53	32631.8	25.2	10_12_2_3	32632.6	0.8
A54	32663.5	34.6	12_12_0_3	32664.6	1.1
A55	32707.1	4.5	11_13_2_2	32706.6	0.5
A56	32737.2	44.7	11_11_1_4	32736.6	0.6
A57	32774.4	6.2	13_11_3_2	32770.6	3.8
A58	32811.6	21.6	12_12_1_3	32810.6	0.9
A59	32848.2	24.6	14_12_3_1	32844.7	3.6
A60	32869.8	4.8	12_13_2_2	32868.7	1.2
A61	32887.8	3.5	13_13_1_2	32884.7	3.1
A62	32922.7	34.7	10_12_2_4	32923.7	1.0
A63	32954.7	11.5	12_12_0_4	32955.7	1.0
A64	32998.0	16.2	11_13_2_3	32997.7	0.3
A65	33028.4	28.5	11_11_1_5	33027.7	0.7
A66	33050.4	3.3	12_11_4_3	33045.7	4.7
A67	33072.5	4.0	12_14_2_2	33071.7	0.7
A68	33102.6	30.2	12_12_1_4	33101.7	0.8
A69	33138.5	8.1	14_12_3_2	33135.7	2.8
A70	33176.8	16.5	13_13_1_3	33175.8	1.0
A71	33213.2	22.7	15_13_3_1	33207.8	5.5
A72	33250.3	3.8	12_12_2_4	33247.8	2.5
A73	33288.0	23.1	16_14_3_0	33283.8	4.1
A74	33320.0	12.6	11_11_3_5	33319.8	0.2
A75	33362.3	13.4	12_14_2_3	33362.8	0.5
A76	33393.8	21.4	12_12_1_5	33392.8	1.0
A77	33467.9	24.3	13_13_1_4	33466.8	1.0
A78	33504.4	8.7	15_13_3_2	33500.9	3.5
A79	33542.5	11.6	14_14_1_3	33540.9	1.6
A80	33578.2	15.7	16_14_3_1	33574.9	3.3
A81	33613.2	4.6	13_13_0_5	33611.9	1.3
A82	33653.0	18.2	12_14_2_4	33653.9	0.9
A83	33685.1	8.2	14_14_2_3	33686.9	1.9
A84	33727.9	11.9	11_13_3_5	33726.0	1.9
A85	33759.2	17.4	13_13_1_5	33757.9	1.3
A86	33833.4	16.8	14_14_1_4	33832.0	1.4
A87	33868.9	7.3	16_14_1_3	33865.0	3.9
A88	33907.0	11.8	13_13_0_6	33903.0	4.0
A89	33944.0	12.4	13_13_0_6	33945.0	1.0
A90	34018.6	14.8	12_14_2_5	34018.0	0.6
A91	34051.1	10.3	13_13_3_5	34050.1	1.0
A92	34125.0	11.1	14_14_1_5	34123.1	2.0
A93	34198.1	9.5	13_13_0_7	34194.1	4.0
A94	34309.0	7.9	13_15_0_6	34309.1	0.2
A95	34599.3	3.5	13_15_2_6	34601.3	2.0
A96	34823.3	2.2	16_13_1_7	34826.3	3.0
A97	34886.3	8.7	16_14_2_6	34884.3	2.0

**Appendix 1—table 5.** Summary of molecular weights (MWs) of B.1.351 receptor binding domain (RBD) glycoforms (Beta, Bx) with respective relative abundances identified by ESI-MS and putative H_N_F_S combinations.

RBD	MeasuredMW (Da)	Relative abundance	H_N_F_S combination	Theoretical MW (Da)	Mass difference (Da)
B1	31247.7	10.6	9_9_0_2	31255.0	7.3
B2	31352.5	18.2	10_8_1_2	31360.0	7.6
B3	31376.3	12.9	9_11_0_1	31370.0	6.2
B4	31408.6	25.9	11_11_0_0	31403.1	5.5
B5	31433.0	16.9	11_9_1_1	31434.0	1.0
B6	31512.7	19.6	9_11_1_1	31516.1	3.4
B7	31538.1	62.3	9_9_0_3	31546.1	8.0
B8	31559.8	7.0	10_9_1_2	31563.1	3.2
B9	31595.6	34.7	9_10_1_2	31604.1	8.6
B10	31616.5	9.9	10_10_0_2	31620.1	3.6
B11	31641.7	11.0	11_10_1_1	31637.1	4.6
B12	31669.2	15.4	13_10_1_0	31670.1	0.9
B13	31700.2	100.0	10_9_0_3	31708.1	7.9
B14	31723.8	64.4	11_9_1_2	31725.1	1.3
B15	31758.7	10.9	10_10_1_2	31766.2	7.5
B16	31778.5	16.2	11_10_0_2	31782.2	3.7
B17	31803.0	12.5	12_10_1_1	31799.2	3.8
B18	31829.5	42.9	10_11_2_1	31824.2	5.3
B19	31886.1	84.9	12_9_1_2	31887.2	1.1
B20	31906.0	32.1	10_10_0_3	31911.2	5.2
B21	31962.0	22.6	10_11_1_2	31969.3	7.2
B22	31991.3	58.7	10_9_2_3	31000.2	8.9
B23	32014.8	31.8	11_9_1_3	32016.2	1.5
B24	32066.4	53.9	11_10_0_3	32073.3	6.9
B25	32089.4	36.3	12_10_1_2	32090.3	0.9
B26	32142.2	10.0	12_11_0_2	32147.3	5.1
B27	32177.0	45.9	12_9_1_3	32178.3	1.3
B28	32196.7	21.8	10_10_0_4	32202.3	5.6
B29	32251.5	48.1	13_10_1_2	32252.3	0.9
B30	32271.1	10.1	11_11_0_3	32276.3	5.3
B31	32326.4	14.9	14_11_1_1	32326.4	0.1
B32	32356.4	33.3	11_10_0_4	32364.4	8.0
B33	32379.5	27.6	12_10_1_3	32381.4	1.9
B34	32431.3	48.8	12_11_0_3	32438.4	7.1
B35	32454.7	29.5	11_9_2_4	32453.4	1.3
B36	32541.6	24.4	13_10_1_3	32543.4	1.8
B37	32560.9	20.8	11_11_0_4	32567.4	6.6
B38	32617.1	43.1	14_11_1_2	32617.5	0.4
B39	32637.1	9.6	12_12_0_3	32641.5	4.4
B40	32722.1	35.2	12_11_0_4	32729.5	7.4
B41	32744.8	17.2	13_11_1_3	32746.5	1.7
B42	32796.8	34.1	11_10_1_5	32801.5	4.7
B43	32820.3	13.6	12_10_2_4	32818.5	1.8
B44	32927.0	9.0	12_12_0_4	32932.6	5.6
B45	32983.0	30.0	13_10_2_4	32980.6	2.4
B46	33109.9	10.9	12_10_2_5	33109.6	0.3
B47	33162.3	26.9	12_11_1_5	33166.6	4.4
B48	33273.5	21.2	13_10_2_5	33271.7	1.8
B49	33348.1	27.5	14_11_2_4	33345.7	2.4
B50	33452.7	24.3	12_11_1_6	33457.7	5.1
B51	33528.0	25.5	13_12_1_5	33531.8	3.8
B52	33637.9	17.3	14_11_2_5	33636.8	1.1
B53	33713.1	20.7	13_10_3_6	33708.8	4.3
B54	33817.9	21.0	13_12_1_6	33822.9	5.0
B55	33893.0	17.0	14_13_1_5	33896.9	3.9
B56	34003.3	13.8	13_10_3_7	33999.9	3.4
B57	34183.0	10.0	14_13_1_6	34188.0	5.0
B58	34548.7	7.7	15_14_1_6	34553.1	4.5
B59	34693.1	13.5	15_14_2_6	34699.2	6.1

**Appendix 1—table 6.** Summary of molecular weights (MWs) of B.1.351.2 receptor binding domain (RBD) glycoforms (Delta, Dx) with respective relative abundances identified by ESI-MS and putative H_N_F_S combinations.

RBD	MeasuredMW (Da)	Relative abundance	H_N_F_S combination	Theoretical MW (Da)	Mass difference (Da)
D1	31073.5	20.9	8_11_1_0	31079.0	5.5
D2	31094.3	1.0	9_11_0_0	31095.0	0.7
D3	31259.9	24.3	10_11_0_0	31257.1	2.8
D4	31363.7	28.6	8_11_1_1	31370.1	6.4
D5	31423.0	23.4	11_11_0_0	31419.1	3.9
D6	31526.2	26.6	9_11_1_1	31532.2	5.9
D7	31548.3	25.4	10_11_0_1	31548.2	0.2
D8	31654.2	29.4	8_11_1_2	31661.2	7.0
D9	31690.5	23.9	10_11_1_1	31694.2	3.7
D10	31711.4	39.3	11_11_0_1	31710.2	1.2
D11	31733.5	16.1	9_12_1_1	31735.2	1.7
D12	31813.8	33.6	10_13_1_0	31809.3	4.5
D13	31838.0	31.0	10_11_0_2	31839.3	1.3
D14	31875.9	13.2	12_11_2_0	31873.3	2.6
D15	31895.2	25.7	10_12_1_1	31897.3	2.1
D16	31979.4	30.5	10_11_1_2	31985.3	5.9
D17	31000.6	60.7	11_11_0_2	31001.3	0.8
D18	32022.0	26.0	9_12_1_2	32026.3	4.3
D19	32079.6	25.4	12_12_0_1	32075.3	4.2
D20	32104.2	49.0	10_13_1_1	32100.4	3.8
D21	32127.5	29.3	10_11_0_3	32130.3	2.8
D22	32164.9	4.6	12_11_0_2	32163.4	1.6
D23	32185.4	44.8	10_12_1_2	32188.4	3.0
D24	32206.8	5.0	11_12_0_2	32204.4	2.4
D25	32290.9	90.6	11_11_0_3	32292.4	1.5
D26	32312.4	32.4	9_12_1_3	32317.4	5.1
D27	32348.4	26.0	11_12_1_2	32350.4	2.1
D28	32369.1	34.1	12_12_0_2	32366.4	2.7
D29	32394.4	45.5	10_13_1_2	32391.5	3.0
D30	32475.9	50.0	10_12_1_3	32479.5	3.5
D31	32496.5	22.2	11_12_0_3	32495.5	1.0
D32	32553.6	0.7	11_13_1_2	32553.5	0.1
D33	32581.7	100.0	11_11_0_4	32583.5	1.8
D34	32603.7	34.7	12_11_3_2	32601.5	2.2
D35	32638.2	0.3	11_12_1_3	32641.5	3.3
D36	32658.4	41.3	12_12_0_3	32657.5	0.8
D37	32683.3	21.8	10_13_1_3	32682.6	0.7
D38	32766.4	41.9	10_12_1_4	32770.6	4.2
D39	32843.0	8.0	11_13_1_3	32844.6	1.7
D40	32873.4	68.5	11_11_0_5	32874.6	1.2
D41	32895.2	18.5	12_11_3_3	32892.6	2.6
D42	32948.2	45.1	12_12_0_4	32948.6	0.4
D43	33238.9	42.5	12_12_0_5	33239.7	0.8
D44	33313.9	30.2	13_13_0_4	33313.8	0.1
D45	33604.6	37.1	13_13_0_5	33604.9	0.2
D46	33896.1	29.5	13_13_0_6	33896.0	0.2
D47	33969.9	9.2	12_12_3_6	33969.0	0.9
D48	34186.4	21.6	13_13_0_7	34187.0	0.7
D49	34260.3	9.0	14_14_0_6	34261.1	0.7

**Appendix 1—table 7.** Summary of molecular weights (MWs) of B.1.1.529 receptor binding domain (RBD) glycoforms (Omicron, Ox) with respective relative abundances identified by ESI-MS and putative H_N_F_S combinations.

RBD	MeasuredMW (Da)	Relative abundance	H_N_F_S combination	Theoretical MW (Da)	Mass difference (Da)
O1	32489.2	40.2	10_11_1_3	32487.6	1.5
O2	32538.4	45.7	11_9_3_3	32535.7	2.7
O3	32559.4	5.6	11_12_1_2	32561.7	2.3
O4	32576.0	23.0	10_10_1_4	32575.7	0.3
O5	32595.1	74.6	11_10_2_3	32592.7	2.4
O6	32615.4	31.4	12_10_1_3	32608.7	6.7
O7	32651.1	29.0	11_11_1_3	32649.7	1.4
O8	32670.2	24.9	12_11_2_2	32666.7	3.4
O9	32702.3	42.5	11_12_0_3	32706.7	4.4
O10	32723.8	87.1	10_10_2_4	32721.7	2.1
O11	32744.0	2.5	11_10_3_3	32738.7	5.3
O12	32761.0	11.3	12_10_2_3	32754.7	6.3
O13	32779.8	53.6	10_11_1_4	32778.7	1.1
O14	32798.7	23.0	11_11_2_3	32795.8	2.9
O15	32829.1	62.1	11_9_3_4	32826.8	2.3
O16	32852.5	27.6	11_12_1_3	32852.8	0.3
O17	32885.7	85.7	11_10_2_4	32883.8	2.0
O18	32905.7	29.7	12_10_3_3	32900.8	4.9
O19	32942.2	12.1	11_11_1_4	32940.8	1.4
O20	32960.8	40.8	12_11_2_3	32957.8	3.0
O21	33014.9	100.0	12_12_1_3	33014.8	0.1
O22	33035.6	25.5	11_10_3_4	33029.8	5.8
O23	33070.2	52.1	12_13_0_3	33071.9	1.6
O24	33089.8	52.7	11_11_2_4	33086.9	2.9
O25	33120.4	52.4	13_11_2_3	33119.9	0.6
O26	33144.7	28.3	11_12_1_4	33143.9	0.8
O27	33177.5	50.7	11_10_2_5	33174.9	2.6
O28	33195.9	33.2	12_10_3_4	33191.9	4.0
O29	33217.4	13.2	12_13_1_3	33217.9	0.5
O30	33233.9	1.6	11_11_1_5	33231.9	2.0
O31	33251.6	58.4	12_11_2_4	33248.9	2.7
O32	33271.2	15.4	13_11_3_3	33265.9	5.2
O33	33306.1	83.2	12_12_1_4	33305.9	0.2
O34	33326.7	28.9	13_12_2_3	33322.9	3.7
O35	33361.5	30.3	12_13_0_4	33362.9	1.4
O36	33380.8	61.4	13_13_1_3	33380.0	0.9
O37	33402.5	0.3	14_13_0_3	33396.0	6.5
O38	33435.9	27.5	11_12_1_5	33435.0	0.9
O39	33454.8	15.1	12_12_2_4	33452.0	2.8
O40	33486.2	24.5	14_12_0_4	33484.0	2.2
O41	33508.3	18.3	12_13_1_4	33509.0	0.7
O42	33542.1	37.0	12_11_2_5	33540.0	2.1
O43	33561.5	24.8	15_13_0_3	33558.0	3.5
O44	33597.5	25.2	12_12_1_5	33597.0	0.5
O45	33617.3	33.1	13_12_2_4	33614.0	3.2
O46	33671.8	47.2	13_13_1_4	33671.1	0.7
O47	33691.7	16.4	14_13_0_4	33687.1	4.6
O48	33726.3	13.7	13_14_0_4	33728.1	1.7
O49	33745.7	35.3	12_12_2_5	33743.1	2.6
O50	33776.2	16.1	14_12_2_4	33776.1	0.1
O51	33800.4	17.0	12_13_1_5	33800.1	0.3
O52	33833.8	3.0	14_13_1_4	33833.1	0.7
O53	33851.7	28.4	15_13_0_4	33849.1	2.6
O54	33874.5	10.3	13_14_1_4	33874.1	0.4
O55	33907.8	21.7	13_12_2_5	33905.1	2.7
O56	33927.2	14.4	14_12_3_4	33922.1	5.0
O57	33963.1	27.1	13_13_1_5	33962.2	0.9
O58	33982.3	13.7	14_13_0_5	33978.1	4.2
O59	34037.0	32.6	14_14_1_4	34036.2	0.8
O60	34058.2	3.7	13_12_3_5	34051.2	7.0
O61	34090.9	8.8	12_13_1_6	34091.2	0.3
O62	34110.9	11.6	13_13_2_5	34108.2	2.7
O63	34142.7	22.5	15_13_0_5	34140.2	2.5
O64	34165.6	5.1	13_14_1_5	34165.2	0.4
O65	34199.3	6.6	15_14_1_4	34198.2	1.1
O66	34217.1	20.9	14_12_1_6	34212.2	4.8
O67	34274.9	20.8	14_13_2_5	34270.3	4.6
O68	34327.5	26.7	14_14_1_5	34327.3	0.2
O69	34346.6	15.5	13_12_3_6	34342.3	4.3
O70	34384.5	0.9	13_10_4_7	34373.3	11.2
O71	34402.4	23.3	13_13_2_6	34399.3	3.1
O72	34457.1	9.0	13_14_1_6	34456.3	0.8
O73	34506.7	18.8	14_12_1_7	34503.3	3.4
O74	34529.7	3.1	12_13_4_6	34529.4	0.3
O75	34564.3	25.0	14_13_0_7	34560.3	4.0
O76	34620.0	26.6	14_14_1_6	34618.4	1.6
O77	34638.4	23.0	15_14_2_5	34635.4	3.0
O78	34658.8	12.7	16_14_3_4	34652.4	6.4
O79	34768.4	0.9	14_14_2_6	34764.4	4.0
O80	34798.6	29.8	14_12_1_8	34794.4	4.2
O81	34840.4	0.5	13_13_3_7	34836.5	4.0
O82	34858.9	20.4	14_13_0_8	34851.4	7.5
O83	35019.8	14.5	15_13_0_8	35013.5	6.3
O84	35041.2	10.5	13_14_3_7	35039.5	1.7
O85	35220.7	23.8	15_14_0_8	35216.6	4.1
O86	35312.3	2.6	15_13_2_8	35305.6	6.7
O87	35354.0	19.8	15_14_1_8	35362.6	8.6

**Appendix 1—table 8.** Summary of molecular weights (MWs) of endoF3-treated WHu-Hu-1 receptor binding domain (RBD) glycoforms (eWTx) with respective relative abundances identified by ESI-MS and putative H_N_F_S combinations.

RBD	MeasuredMW (Da)	Relative abundance	H_N_F_S combination	Theoretical MW (Da)	Mass difference (Da)	Number of trimmed N-glycans
eWT1	28116.9	4.5	1_3_0_0	28123.9	7.0	2
eWT2	28243.9	26.4	3_2_0_0	28244.9	1.0	2
eWT3	28357.3	14.5	1_2_1_1	28358.0	0.6	2
eWT4	28618.0	23.8	1_4_2_0	28619.1	1.0	2
eWT5	28881.1	2.4	3_3_1_1	28885.1	4.0	2
eWT6	29029.2	18.7	3_3_2_1	29031.2	2.1	2
eWT7	29143.8	1.1	6_4_0_0	29137.2	6.5	1
eWT8	29318.2	1.1	5_5_1_0	29324.3	6.1	1
eWT9	29406.5	8.2	5_4_1_1	29412.3	5.8	1
eWT10	29454.5	14.1	4_5_1_1	29453.4	1.2	1
eWT11	29649.4	0.6	7_5_1_0	29648.4	1.0	1
eWT12	29736.3	9.6	5_7_1_0	29730.5	5.8	1
eWT13	29753.5	1.3	5_5_0_2	29760.5	6.9	1
eWT14	30084.9	1.3	7_5_2_1	30085.6	0.7	1
eWT15	30103.4	10.3	5_6_1_2	30109.6	6.2	1
eWT16	31033.0	27.6	8_6_2_3	31032.9	0.1	1
eWT17	31216.4	17.2	7_7_1_4	31219.0	2.5	1
eWT18	31416.8	8.0	7_8_1_4	31422.0	5.2	1
eWT19	31437.4	0.9	8_8_0_4	31438.0	0.7	1
eWT20	31482.3	9.0	7_9_2_3	31480.1	2.2	1
eWT21	31505.7	38.4	9_9_0_3	31512.1	6.4	0
eWT22	31543.3	41.0	9_7_1_4	31543.1	0.2	1
eWT23	31564.8	55.2	7_8_2_4	31568.1	3.3	1
eWT24	31607.4	2.2	11_10_3_0	31604.1	3.2	0
eWT25	31644.1	27.3	8_9_2_3	31642.1	2.0	1
eWT26	31668.2	82.8	10_9_0_3	31674.1	5.9	0
eWT27	31691.0	33.8	11_9_1_2	31691.1	0.2	0
eWT28	31708.4	9.9	9_10_0_3	31715.2	6.7	0
eWT29	31727.6	58.6	10_10_1_2	31732.2	4.5	0
eWT30	31748.3	42.7	11_10_0_2	31748.2	0.1	0
eWT31	31770.9	29.2	12_10_3_0	31766.2	4.7	0
eWT32	31795.9	25.7	10_11_0_2	31789.2	6.7	0
eWT33	31833.6	35.0	11_9_2_2	31837.2	3.6	0
eWT34	31854.4	95.9	9_10_1_3	31861.2	6.8	0
eWT35	31872.6	14.1	10_10_2_2	31878.2	5.6	0
eWT36	31893.5	1.5	11_10_1_2	31894.2	0.7	0
eWT37	31912.9	51.6	12_10_0_2	31910.2	2.6	0
eWT38	31933.2	26.1	10_11_1_2	31935.3	2.0	0
eWT39	31958.1	100.0	11_11_0_2	31951.2	6.9	0
eWT40	31980.4	28.5	13_11_0_1	31984.3	3.9	0
eWT41	32017.7	36.8	10_10_1_3	32023.3	5.5	0
eWT42	32060.9	6.2	12_10_1_2	32056.3	4.6	0

**Appendix 1—table 9.** Summary of molecular weights (MWs) of endoF3-treated B.1.351.2 receptor binding domain (RBD) glycoforms (eDx) with respective relative abundances identified by ESI-MS and putative H_N_F_S combinations.

RBD	MeasuredMW (Da)	Relative abundance	H_N_F_S combination	Theoretical MW (Da)	Mass difference (Da)	Number of trimmed N-glycans
eD1	28681.8	2.2	4_3_0_0	28680.1	1.7	2
eD2	28720.3	24.9	3_4_0_0	28721.1	0.8	2
eD3	28741.8	35.3	2_2_2_1	28736.1	5.6	2
eD4	28762.6	9.4	2_5_0_0	28762.2	0.5	2
eD5	28782.3	33.5	1_3_2_1	28777.2	5.2	2
eD6	28803.6	25.9	3_3_2_0	28810.2	6.5	2
eD7	28826.2	19.8	4_3_1_0	28826.2	0.1	2
eD8	28867.3	82.4	3_4_1_0	28867.2	0.1	2
eD9	28888.2	52.6	4_4_0_0	28883.2	5.0	2
eD10	28907.6	20.6	3_2_2_1	28898.2	9.4	2
eD11	28927.8	100.0	3_5_0_0	28924.2	3.6	2
eD12	28949.2	57.5	3_3_1_1	28955.2	6.0	2
eD13	28970.6	38.3	4_3_0_1	28971.2	0.6	2
eD14	28990.7	30.3	2_4_1_1	28996.2	5.5	2
eD15	29011.6	32.3	3_4_0_1	29012.2	0.6	2
eD16	29032.7	24.1	4_4_1_0	29029.2	3.5	2
eD17	29053.6	52.1	3_2_1_2	29043.2	10.4	2
eD18	29074.7	51.9	3_5_1_0	29070.3	4.4	2
eD19	29095.1	15.7	3_3_2_1	29101.3	6.2	2
eD20	29114.2	37.7	4_3_1_1	29117.3	3.1	2
eD21	29135.1	26.0	2_4_0_2	29141.3	6.2	2
eD22	29158.3	34.9	3_4_1_1	29158.3	0.0	2
eD23	29179.7	25.3	4_4_2_0	29175.3	4.4	2
eD24	29198.8	17.6	3_2_2_2	29189.3	9.6	2
eD25	29219.2	43.7	3_5_0_1	29215.3	3.9	2
eD26	29240.2	24.3	3_3_1_2	29246.3	6.1	2
eD27	29261.4	16.4	4_3_2_1	29263.3	1.9	2
eD28	29281.5	6.4	2_4_1_2	29287.3	5.9	2
eD29	29301.3	1.9	3_4_2_1	29304.3	3.0	2
eD30	29302.0	1.2	3_4_0_2	29303.3	1.3	2
eD31	29344.9	24.8	5_4_2_0	29337.4	7.6	2
eD32	29366.8	5.0	3_5_1_1	29361.4	5.5	2
eD33	29405.7	10.1	4_3_1_2	29408.4	2.7	2
eD34	29427.1	3.8	2_4_2_2	29433.4	6.3	2

**Appendix 1—table 10.** Glycopeptide analysis of wild-type (WT) receptor binding domain (RBD).

N-site	Peptide sequence	N-glycan	Retention time (min)	Measured m/z	Charge state	Mass accuracy (ppm)	MS area
N331	PNITNLCPFGEV	A2S2	18.0	1170.471	3	–3.66	88472
N331	PNITNLCPFGEV	A2S2	18.0	1755.705	2	–2.48	70771
N331	PNITNLCPFGEV	A1G1F	18.2	1354.577	2	0.45	157724
N331	PNITNLCPFGEV	A1G0F	18.3	1273.551	2	0.14	85745
N331	PNITNLCPFGEV	A1S1F	18.6	1500.124	2	0.04	65793
N331	FPNIT	A2G2B	27.8	1209.489	2	–2.73	57370
N343	GEVFNATR	A2S1G1FB	31.0	1579.132	2	–3.43	44341
N343	NATRF	A2G2F	6.3	1189.484	2	0.33	107145
N343	NATRF	A2G1F	6.5	1107.955	2	0.30	46566
N343	NATRF	A2S1G1F	7.0	1335.032	2	0.75	38217
N343	PFGEVFNATR	A2G2	13.5	1381.076	2	–3.29	272764
N343	PFGEVFNATR	A2G1	13.6	1300.049	2	–3.69	150239
N343	PFGEVFNATR	A2S1G1	13.7	1526.623	2	–2.90	305875
N343	PFGEVFNATR	A2S1G0	13.8	1445.597	2	–3.53	164686

**Appendix 1—table 11.** Glycopeptide analysis of endoF3-treated wild-type (WT) receptor binding domain (RBD).

N-site	Peptide sequence	N-glycan	Retention time (min)	Measured m/z	Charge state	Mass accuracy (ppm)	MS area
N331	PNITNLCPFGEVF	A1G0F	18.9	1347.585	2	–3.88	21591
N331	PNITNLCPFGE	A2G0FB	19.5	1427.087	2	–5.81	83634
N331	PNITNLCPFGEV	A1G1F	19.6	1354.575	2	–1.09	112872
N331	PNITNLCPFGEV	A1G0F	19.7	1273.551	2	–1.21	52498
N331	PNITNLCPFGEV	A1S1F	19.9	1500.122	2	–1.51	59378
N331	PNITNLCPF	A2G1	26.2	827.350	3	1.33	49111
N331	PNITNLCPF	A2G1	26.9	1240.524	2	2.81	25854
N331	PNITNL	A1G1	28.6	964.915	2	0.19	19156
N343	NATRF	A2G2F	6.3	1189.483	2	0.54	32460
N343	NATRF	A2G1F	6.4	1107.955	2	0.19	47037
N343	NATRF	A2G0F	6.5	1027.430	2	0.50	23831
N343	NATRF	A2S1G1F	7.5	1335.032	2	0.75	34621
N343	NATRF	A2S2F	7.8	1480.580	2	1.09	24974
N343	NATRF	GnF	8.2	957.453	1	0.28	17273
N343	NATRF	GnF	8.2	479.230	2	0.53	255747
N343	NATR	A2G0	18.0	880.363	2	–2.3	34690
N343	NATRFASVY	A2S1G1	9.4	736.560	4	4.72	103643
N343	GEVFNATR	GnF	11.3	621.797	2	0.83	14586
N343	GEVFNATR	GnF	15.5	414.868	3	3.39	15706
N343	GEVFNATR	GnF	15.7	414.868	3	3.78	18198
N343	GEVFNATRF	GnF	17.6	695.331	2	1.23	17392
N343	GEVFNATRF	A2G0F	23.2	1243.537	2	4.76	17779
